# MicrobeDiscover: A Knowledge Graph–Enabled AI Framework for Identifying Microbes for Inorganic Nanomaterial Biosynthesis

**DOI:** 10.1002/advs.202520587

**Published:** 2026-01-25

**Authors:** Ludi Wang, Hexing Han, Yufeng Liu, Zhiyuan Ning, Yujie Ma, Haidan Wang, Jinling Xu, Qiansheng Huang, Wenjuan Cui, Yuanchun Zhou, Yang Gao, Bin Wang, Yi Du

**Affiliations:** ^1^ Computer Network Information Center Chinese Academy of Sciences Beijing China; ^2^ University of Chinese Academy of Sciences Beijing China; ^3^ Hangzhou Institute for Advanced Study UCAS Hangzhou China; ^4^ CAS Key Laboratory of Nanosystem and Hierarchical Fabrication National Center for Nanoscience and Technology (NCNST) Beijing China; ^5^ Institute of Urban Environment (IUE) Chinese Academy of Sciences Xiamen China; ^6^ School of Ecology and Environment Xizang University Lhasa China

**Keywords:** AI for Science, knowledge graph, microbial synthesis, nanomaterials

## Abstract

As an environmentally friendly, mild, sustainable preparation method for nanomaterials (NMs) and the foundation of microbe‐material hybrid systems, microbial synthesis of NMs hold great promise in the sustainable future. However, reported instances cover only approximately 400 microbes and 90 NMs, merely scratching the surface of the theoretical potential of enormous microbe–NMs combinations. Research methods predominantly based on empirical approaches and trial‐and‐error are significantly challenged in terms of screening efficiency. Here, we introduce an AI‐based framework, MicrobeDiscover, which identifies potential microbes for NM synthesis within a vast search space by integrating and representing microorganisms, NMs, and their interactions. A central component of MicrobeDiscover is a knowledge graph guided by expert insights into microbial synthesis, bridging microbiological and materials science domains to provide the AI model with robust data for screening and predictive modeling. Among the top 20 microorganisms predicted by MicrobeDiscover, the recommendation success efficiency reached 80.77%. Leveraging the framework's predictions, we successfully synthesized several kinds of trimetallic NMs using *Shewanella oneidensis* MR‐1 as suggested by MicrobeDiscover, in the context of non‐trimetallic NMs were reported producing in a biosynthetic way. This approach is anticipated to significantly advance the development of effective microorganisms and enhance the controllable synthesis of NMs.

## Graphical abstract

Microbial synthesis of nanomaterials (NMs) is eco‐friendly, but the screening of microorganisms is limited by inefficient traditional methods (currently only involving∽400 microorganisms/90 NMs). We propose AI framework MicrobeDiscover, integrating a knowledge graph of microbe‐NM interactions. It achieves 80.77% top‐20 recommendation success and enables novel trimetallic NMs synthesis, advancing NM development.

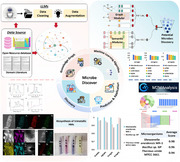

## 1 Introduction

Nanomaterials (NMs), especially inorganic metal NMs, such as single, bimetallic, and trimetallic NMs are widely applied in chemistry, energy, electronics, and biomedical fields. Traditional physical/chemical NMs synthesis methods require harsh conditions and high energy consumption. Whereas microorganisms form NMs by depositing and assembling ions/molecules, which is an eco‐friendly, mild, and sustainable strategy [1, 2, 3, 4, 5]. The emerging microbe–material hybrid systems further enhance their potentials in ${\mathrm{CO}}_{2}$ conversion, hydrogen production, nitrogen fixation, and electron transfer through synergistic effects [6, 7, 8, 9, 10, 11].

To date, microorganisms from bacteria, archaea, and eukaryotes all demonstrate NM synthesis capacity, and have yielded diverse materials, including metal oxides [12, 13], chalcogenides [14, 15], and non‐metallic materials [16, 17]. Despite NCBI recording 63 677 archaeal, bacterial, and fungal species (with many more undiscovered), only hundreds have been explored for NM synthesis [18]. There are perhaps a large number of such microorganisms remain waiting to be studied. However, due to the virtually limitless combinations of microorganisms and NMs, it is impractical to experimentally investigate all potential instances of microbial NMs synthesis.

In addition, the limitation of the synthesis mechanism researches (about 68 research papers as we found till 2024) hinders the discovery of microorganisms using genetic comparisons [1]. Therefore, there is an urgent need for a method to recommend and mine microbial species capable of synthesizing NMs from a vast number of microorganisms, breaking current limitations and expanding the scope of research.

To address above issues, the required method should leverage the existing, albeit limited, knowledge of microbial synthesis of NMs and integrate the extensive knowledge from the fields of microbiology and materials science. This method should be capable of fusing these diverse knowledge bases effectively—a process for which AI is particularly well‐positioned to leverage its analytical capabilities. In the materials and microbiology field, the scientific research empowered by AI for Science [19] has emerged [20, 21, 22, 23, 24, 25, 26, 27]. However, comprehensive exploration in microbial NM synthesis remains limited due to three main factors: (1) It spans microbiology, chemistry and materials science, hindering effective knowledge integration without a unified system; (2) Integration of complex multi‐disciplinary data challenges high‐quality dataset construction for domain applications; (3) Complex interactions between biological mechanisms, chemical reactions and material properties, combined with a large search space, make conventional methods inefficient. Consequently, knowledge–data integration approaches, valued in other fields, are underutilized here, which undoubtedly limits the application of AI in the interdisciplinary field of microbiology, chemistry, and materials science.

As a structured semantic framework that models domain knowledge as an interconnected graph‐based data structure, knowledge graph can model domain knowledge as an interconnected graph‐based data structure, where nodes represent distinct entities and edges encode explicit semantic relationships between these entities. This structure can transform fragmented, unstructured, or semi‐structured information into a machine‐interpretable and logically consistent knowledge base.The microbial synthesis of NMs is influenced by multiple factors, particularly the inherent characteristics of the microorganisms involved. Furthermore, the properties of the synthesized materials cannot yet be fully predicted. This process encompasses various entities, including phylogenetic information of microorganisms, synthesis mechanisms of nanomaterials, precursors, synthesis locations of nanoparticles, and synthesis conditions. As a powerful tool for integrating multi‐source heterogeneous data, knowledge graphs significantly enhance research efficiency and precision by consolidating disorganized and fragmented biological data into a structured and interconnected knowledge network. Whether it is the relationships among microorganisms or the elemental relationships between materials, knowledge graphs can effectively represent them, thereby enhancing the application of artificial intelligence in this field. By leveraging the capabilities of the domain knowledge graph, we developed MicrobeDiscover, an AI‐driven framework incorporating domain knowledge to identify potential microbes for biosynthesis, and applied here for NMs synthesis. Comprising a knowledge graph construction module and a microbial prediction module, MicrobeDiscover first annotates knowledge from 536 literature sources on microbial NM synthesis to form an initial system covering microorganisms, NMs, and synthesis methods. It bridges microbiology and materials science by integrating microbial phylogenetic and phenotypic data from NCBI with materials science elemental composition and property data, using large language models (LLMs) for data alignment and augmentation. The resulting comprehensive knowledge graph includes 415 reported microorganisms, 12 558 potential microbes connected via NCBI, and 87 NMs composed of 36 elements. In the prediction module, MicrobeDiscover employs a hybrid network to convert multi‐source features into unified vector representations, integrating semantic and cross‐domain information. AI‐based microbial representations effectively capture NM correlations for potential microbe discovery. Among the top 20 predictions, 80.77% of them have been experimentally validated in recent literature (post‐2019, which was not included in the training set), showed successful NM biosynthesis. Guided by MicrobeDiscover, we synthesized novel trimetallic NMs (AuPdPt, AgPdPt, AuAgPt, AuAgPd) using *Shewanella oneidensis* MR‐1. This framework enhances NM design and biosynthesis, expanding inorganic NM diversity and scalability. It can also be extended to other materials with new datasets, accelerating eco‐friendly biosynthesis discovery.

## 2 Overview of MicrobeDiscover

**FIGURE 1:**
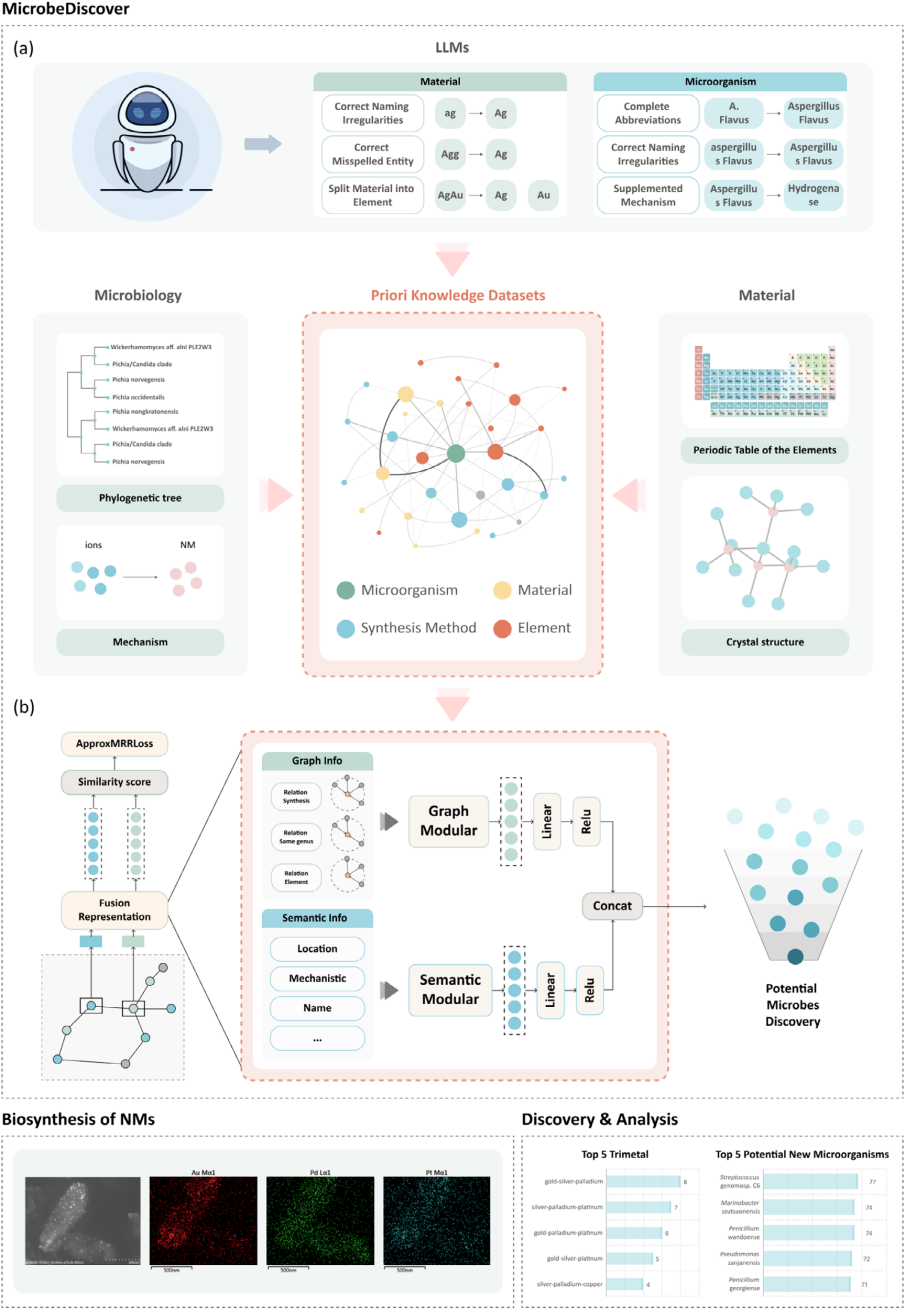
Architecture and prediction process of MicrobeDiscover, our generative AI framework for discovering novel microbes: (a) Knowledge graph construction module; (b) Microbial NM prediction module.

MicrobeDiscover improves potential microbes discovery by incorporating novel neural network architectures and training procedures based on domain knowledge datasets. To convert multi‐source features to unified vector representations, we presented a fusion model consists of Semantic Modular and Graph Modular. The overview of our model is as shown in Figure 1. As shown in Figure 1a, MicrobeDiscover developed an ontology consisting of 21 categories, and annotated knowledge using the screened scientific and technological literature based on it. At the same time, we have utilized a large model to correct deficiencies and errors, thus forming a domain knowledge graph. Furthermore, we have associated the microbial data from NCBI with this knowledge graph based on the phylogenetic tree, resulting in the formation of the Priori Knowledge Dataset. To better explore potential microorganisms, MicrobeDiscover used Graph Modular and Semantic Modular to represent knowledge and predict potential microorganisms, and conduct actual preparation experiments (Figure 1b).

### 2.1 Domain Knowledge Infusion

**FIGURE 2:**
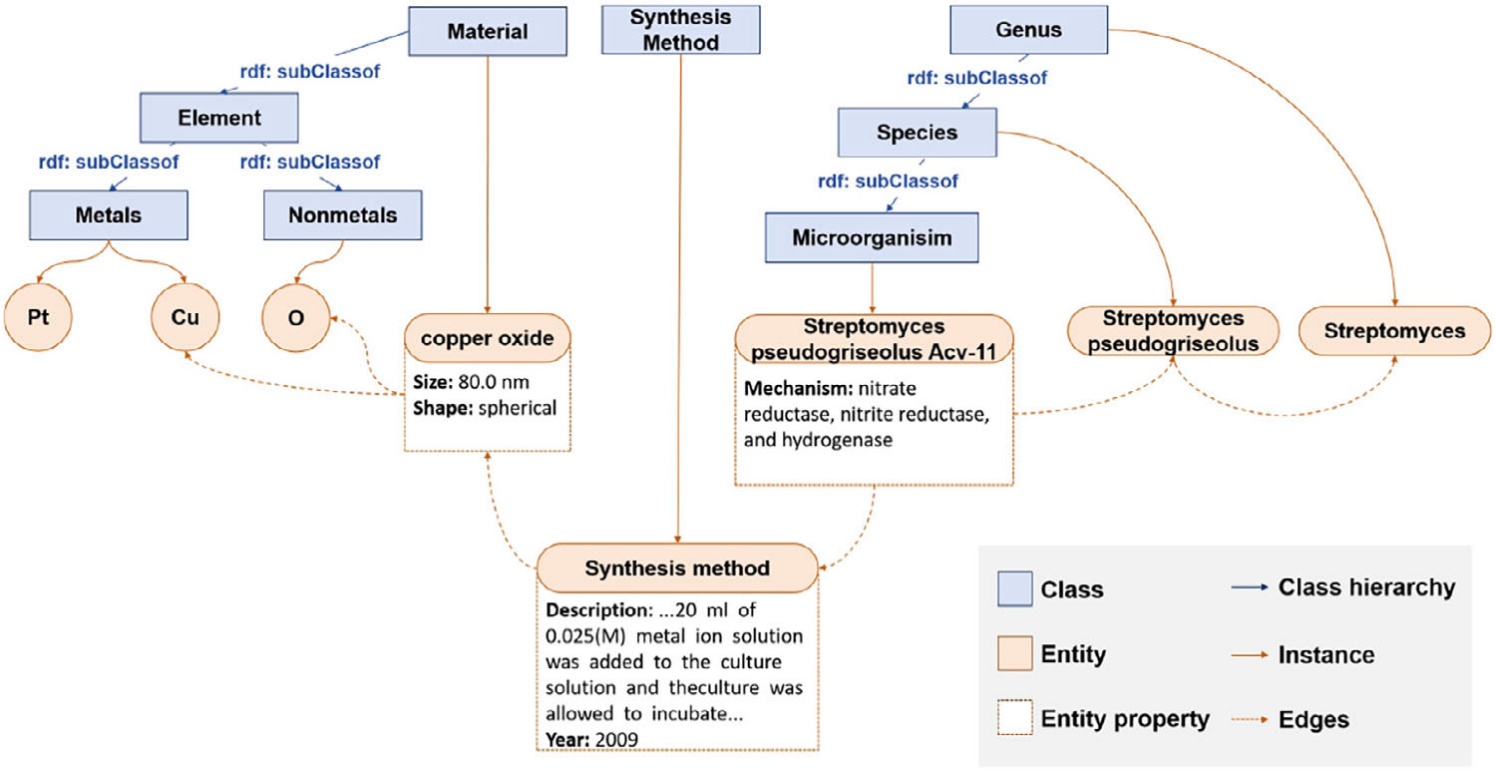
The schematic illustration of knowledge graph structure in MicrobeDiscover.

Figure 2 shows the detailed schematic illustration of the knowledge graph structure. To clarify the knowledge graph's architecture, we differentiate “class level” and “instance level.” At the class level, hierarchies are formed via rdfs:subClassOf: *Element* branches into *Metals* and *Nonmetals*, *Genus* into Species then Microorganism, with Material and Synthesis Method as independent classes. At the instance level, entities (e.g., Pt, Cu, O as elemental instances; copper oxide with size/shape properties; *Streptomyces* taxa as microbial instances; a Synthesis method with procedural/year properties) are linked to classes via rdf:type. Dotted edges encode relations (e.g., material–element composition, entity‐synthesis method associations), enabling systematic domain knowledge organization for interdisciplinary reasoning in materials and microbiology research.

**FIGURE 3:**
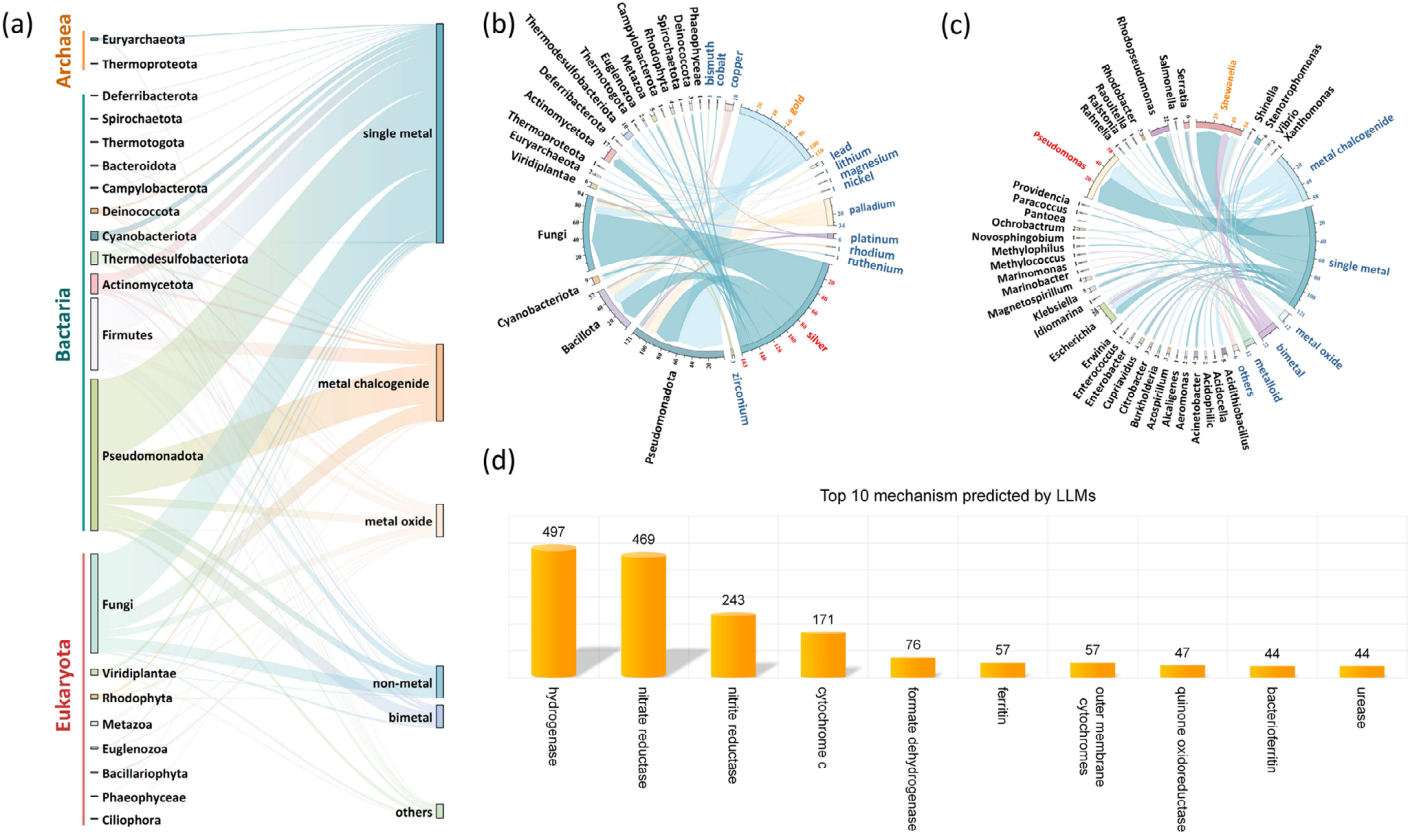
Quantitative analysis of Prior Knowledge Datasets: (a) The relationships between the labelled microorganisms and NMs; (b) The relationship between single metals and microorganisms; (c) The relationship between microbial genera in the phylum Pseudomonadota and NMs;(d) The top 10 mechanisms completed by LLMs.

**FIGURE A1:**
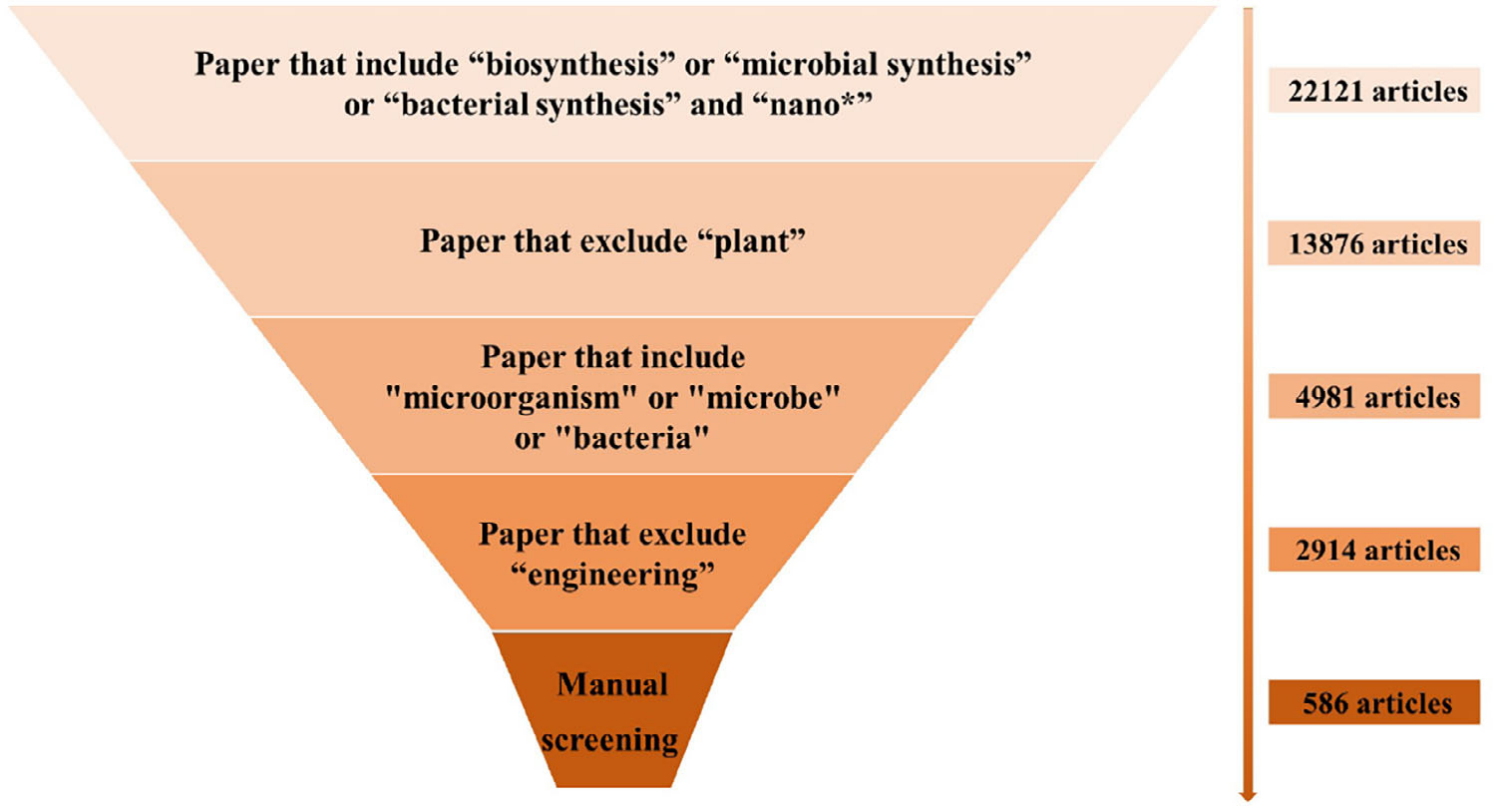
The process of literature screening.

**FIGURE A2:**
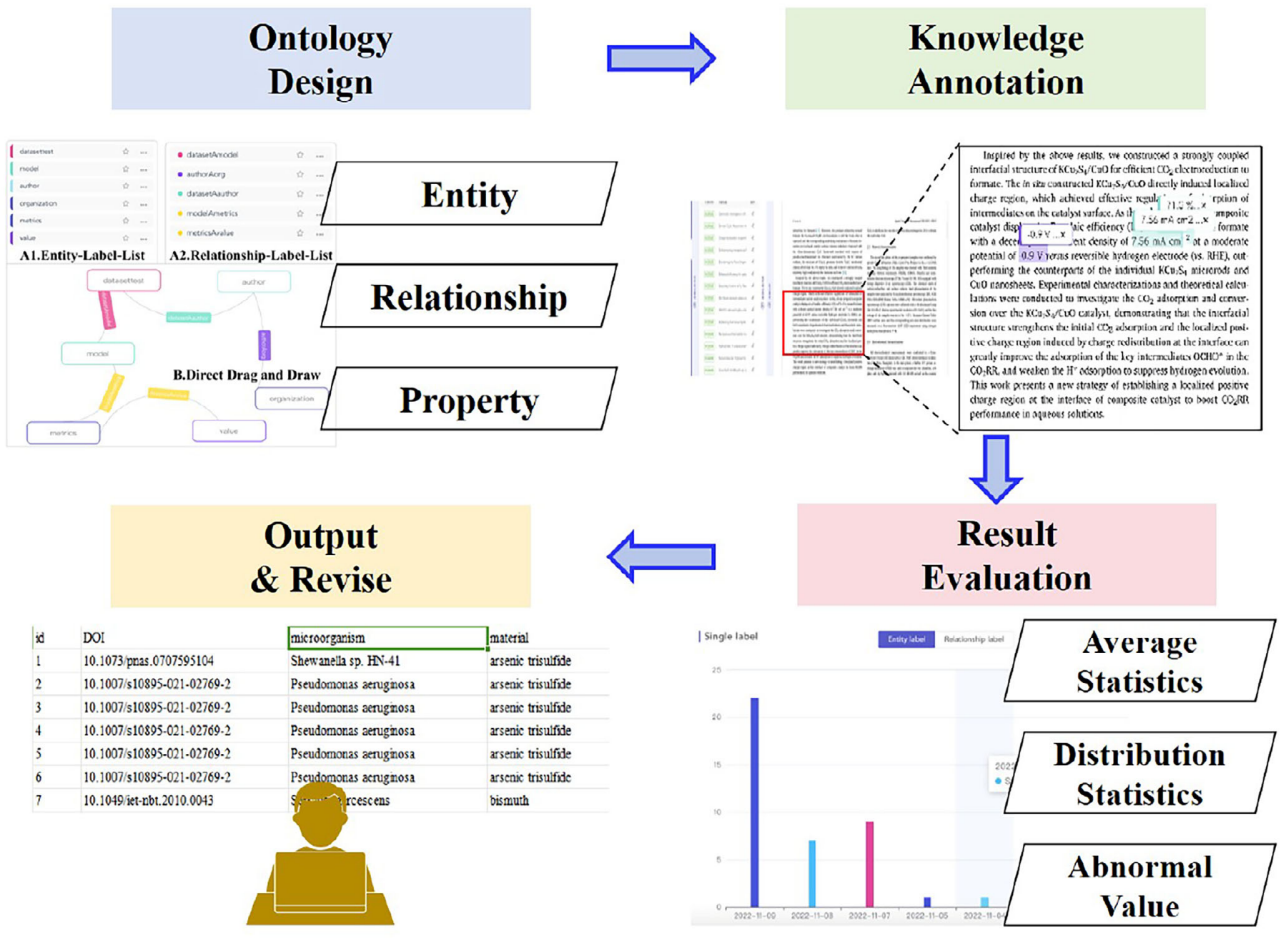
The workflow of data annotation.

The complex interactions between organic microbial systems and inorganic NMs highlight a gap in cross‐disciplinary knowledge integration. To address this, we invited domain experts to extract knowledge data from a total of 536 scientific literature in the field of microbial synthesis of NMs, including microorganisms, precursors, materials, synthesis parameters, and other categories, and used it to associate open source data in related fields to form a domain knowledge database. The details of entities are in Table S1. Figure A1 and A2 presents the process of literature screening and data annotation. We also utilized the summarization and induction ability of LLMs to clean and enhance the extracted knowledge dataset. The domain knowledge dataset can be accessed by 10.57760/sciencedb.10875 [28], and the construction details are in the section of Methods. The quantitative analysis of Prior Knowledge Datasets is as shown in Figure 3.

**FIGURE 4:**
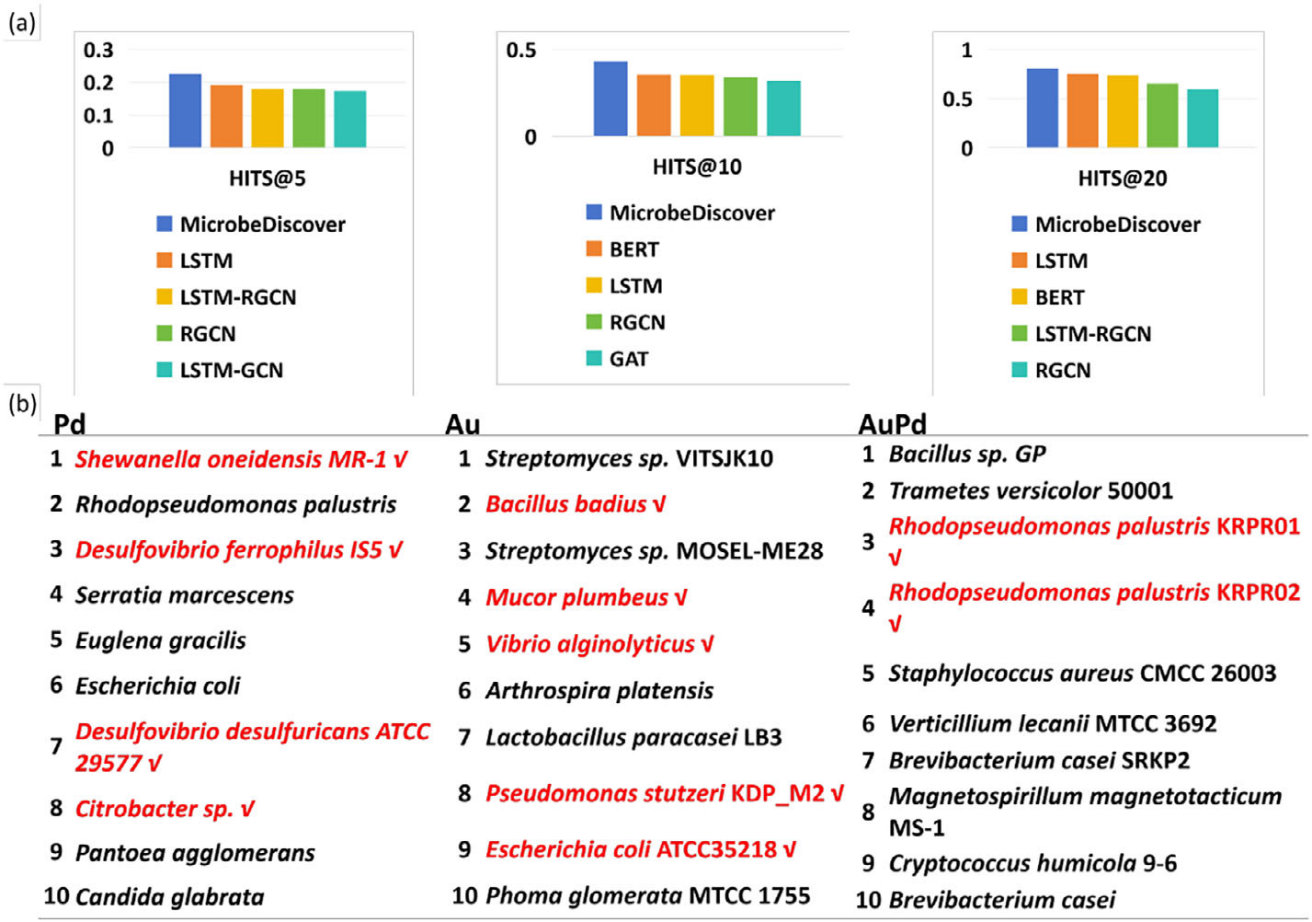
The overview of result and representation visualization: (a) The comparative experimental results, which indicate MicrobeDiscover performs best; (b) Ranking of potential microbes for biosynthesis of Pd, Au, and AuPd, and highlight microbes are reported in 2019‐post studies (which are not used for training).

**FIGURE 5:**
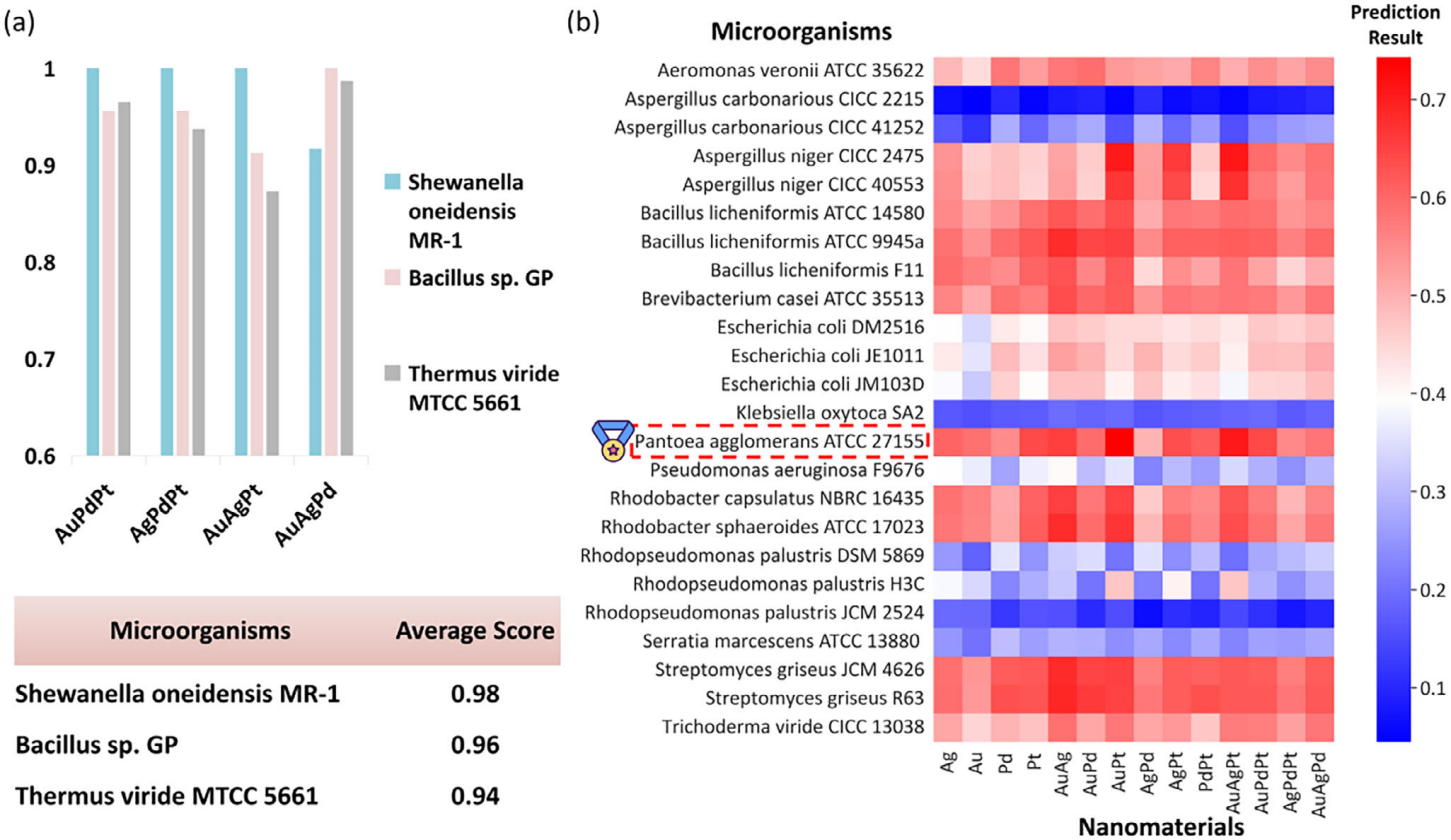
The ability of microorganisms in trimetallic NMs synthesis. (a) The average score and detailed prediction result of top 3 potential microbes, which are *Shewanella oneidensis* MR‐1, *Bacillus* sp. GP and *Thermus viride* MTCC 5661. (b) The predict results of microorganisms that are available and also have not been reported to be capable of synthesizing NMs, where *Pantoea agglomerans* ATCC 27155, *Aspergillus niger* CICC 2475 and *Streptomyces griseus* R63 demonstrate greater potential.

**FIGURE 6:**
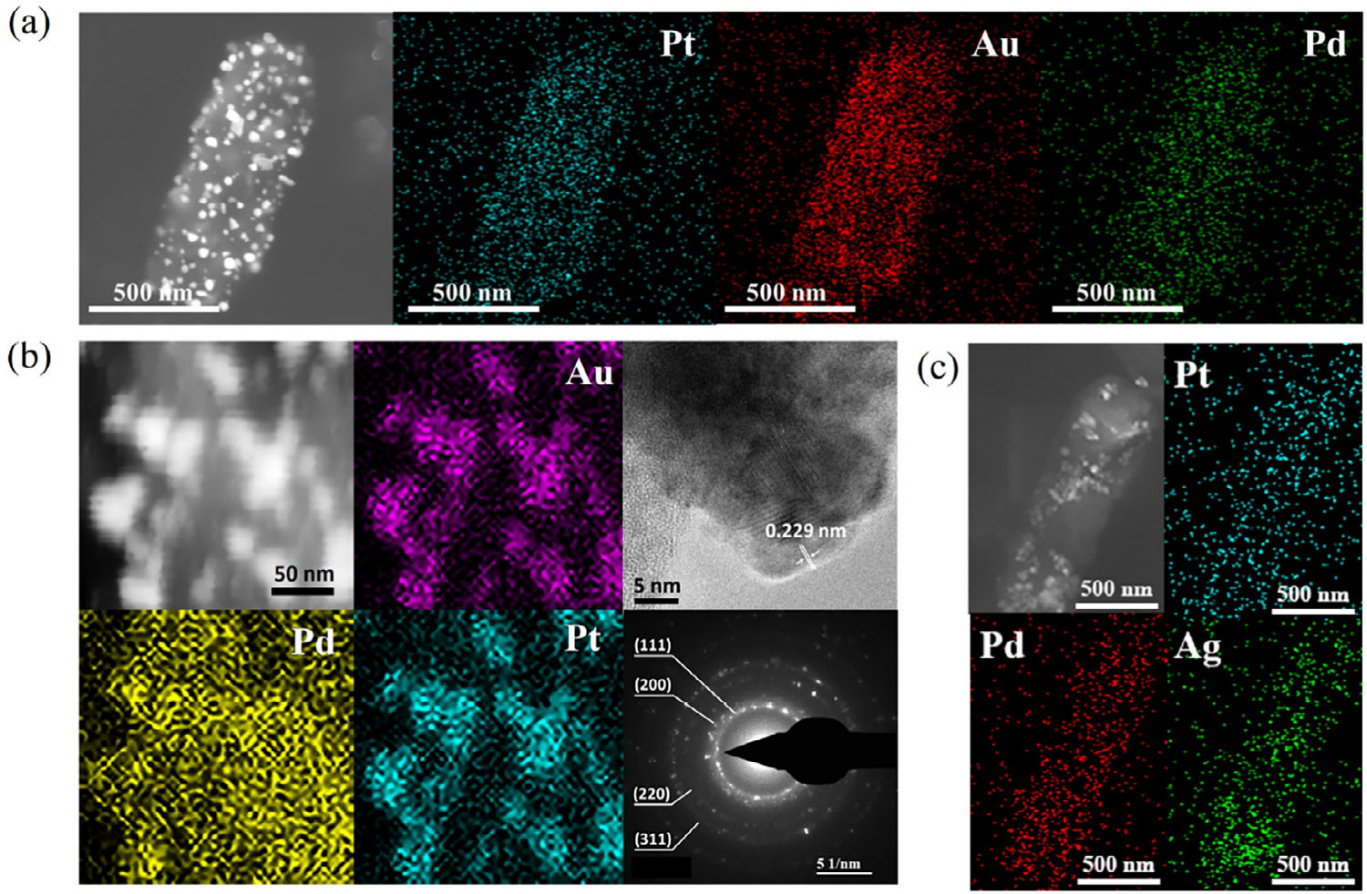
(a) SEM and EDS mapping images of *Shewanella oneidensis* MR‐1, after the synthesis of PtAuPd trimetallic NMs. (b) High‐angle annular dark‐field scanning transmission electron microscopy (HAADF‐STEM) and EDS mapping images of AuPdPt on the cell of *Shewanella oneidensis* MR‐1, HR‐TEM images of the AuPtPd nanoparticles and the SAED pattern. (c) SEM and EDS mapping images of *Pantoea agglomerans* ATCC 27155, after the synthesis of PtPdAg trimetallic NMs.

**FIGURE 7:**
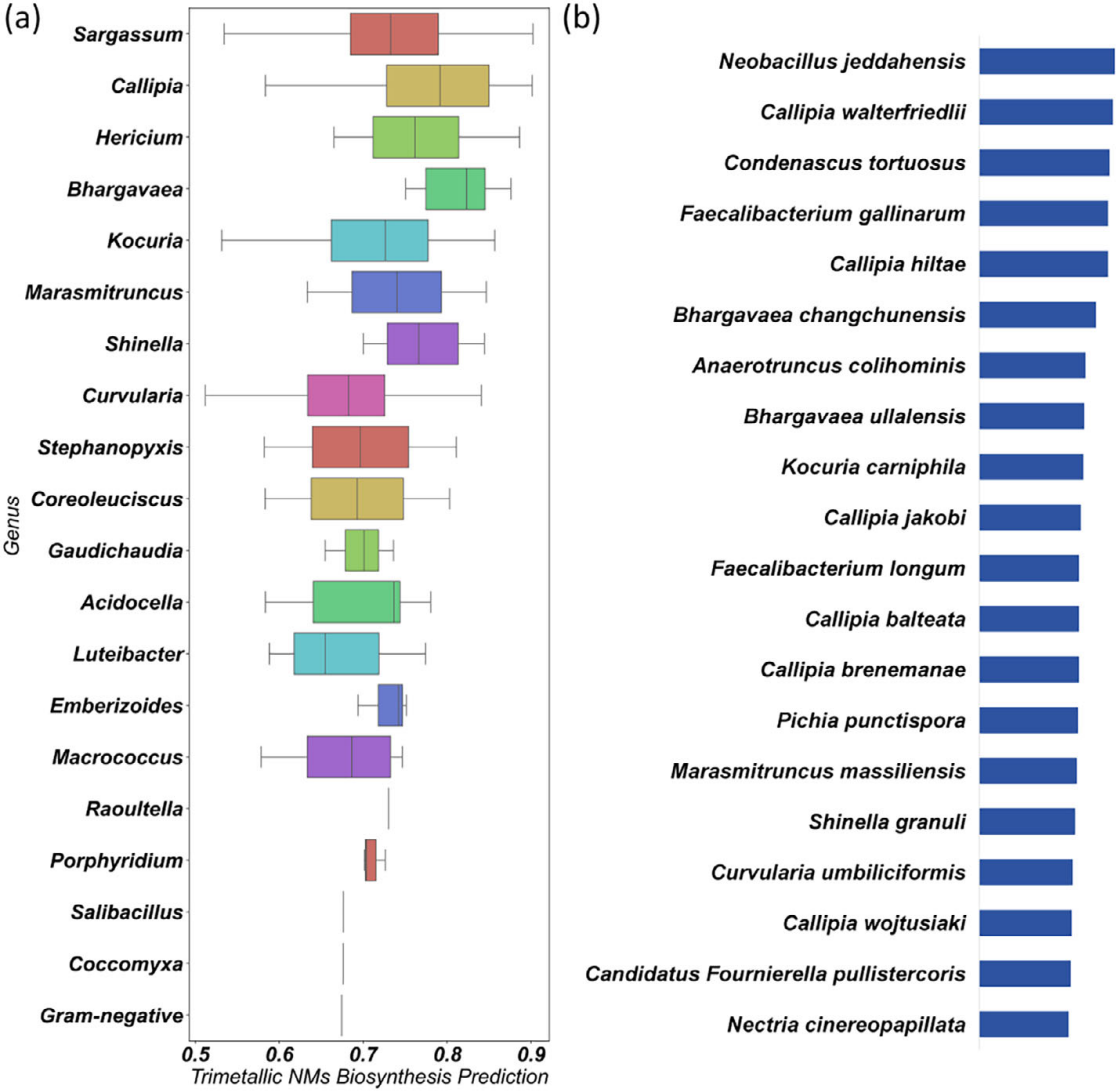
(a) Trimetallic NMs Biosynthesis Prediction Result of Genus from NCBI (Top 20). (b) Top 20 valuable novel microbe species from NCBI.

**FIGURE 8:**
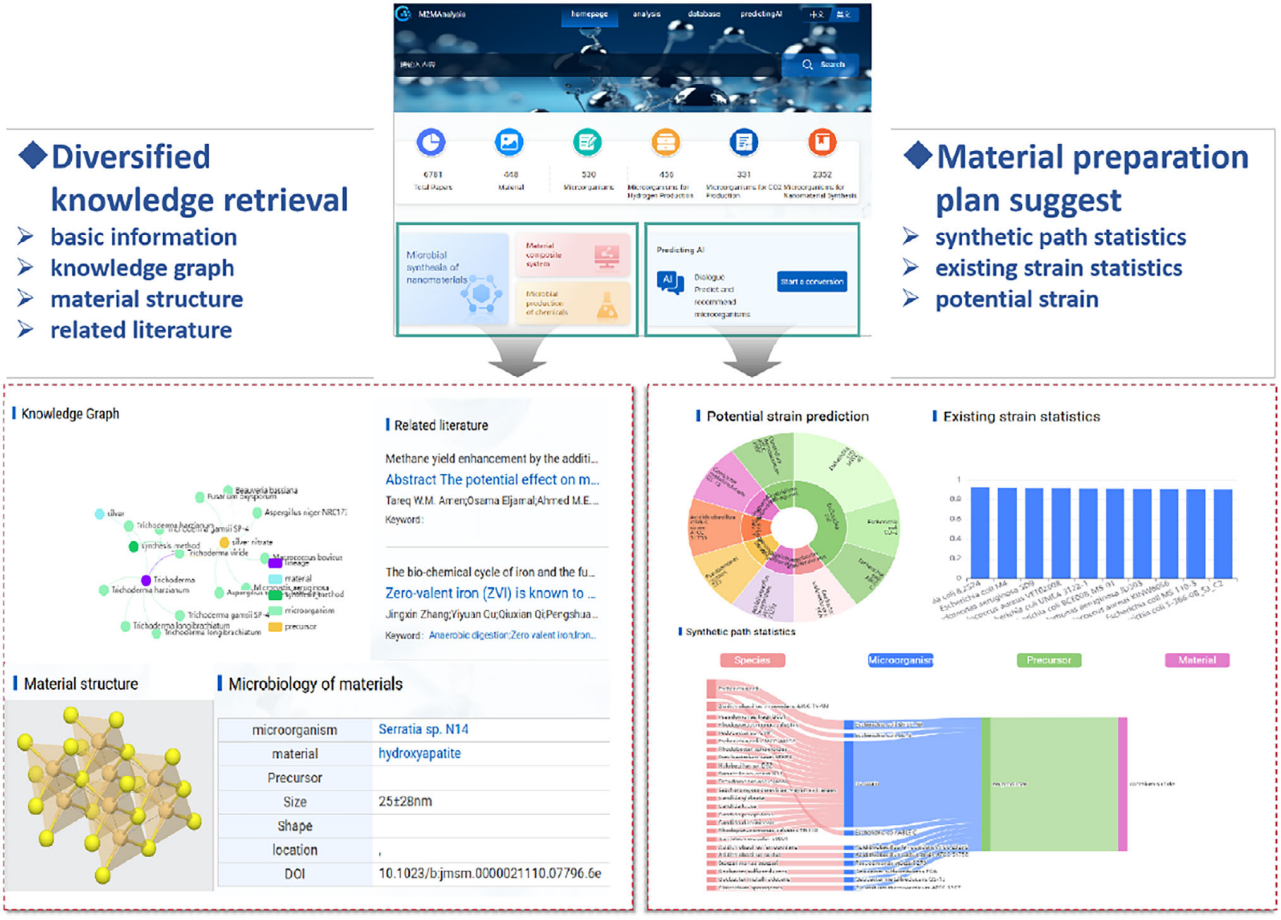
The overview of the online application.

There were 228 species in the labelled literature with a clear phylogenetic tree in NCBI, and they belonged to 21 microbial phylums which distributed in the three domains of life, Archaea, Bacteria, and Eukaryotes, as shown in Figure 3a. Most researches have been done on the synthesis of NMs employing Firmicutes, Pseudomonadota in bacteria, and Fungi in eukaryotes. The Firmicutes (phylum) is composed of Gram‐positive bacteria. They have a peptidoglycan‐containing hard or semi‐rigid cell wall. Certain species possess flagella for movement and are able to generate endospores [29]. Pseudomonadota is a major phylum of Gram‐negative bacteria reported to be dominant in the natural environment and possesses great versatility [30]. Fungi kingdom consists of a hyper‐diverse community of heterotrophic eukaryotes characterized by chitinous cell walls, loss of phagocytosis, and cellular organization ranging from completely unicellular unipolar organisms to highly complex syncitial filaments that can form macroscopic structures [31]. The majority of these microbes have been utilized to manufacture single metals and metal dichalcogenides. In comparison, there were fewer studies on microbial synthesis of metal oxides, bimetals, and non‐metals.

A comprehensive analysis of the single metal is conducted in Figure 3b. Single metals include alkali metals, alkaline earth metals, and transition metals, with a total of 14 kinds. Among these metals, silver emerged as the most extensively studied due to the extensive researches in antibacterial application [32], followed by gold. Fungi kingdom was observed to play a pivotal role in the production of silver. On the other hand, the microorganisms that contributed most significantly to the synthesis of gold belonged to the phylum Pseudomonadota. Further analysis of most studied Pseudomonadota revealed that 40 genera of microorganisms in this phylum were involved in the synthesis of NMs (Figure 3c). Among these genera, *Pseudomonas* and *Shewanella* stand out. *Pseudomonas* synthesized mostly single metals and metal dichalcogenides. *Shewanella* was mostly capable of synthesizing single metals, although it can also produce bimetal, metal dichalcogenides, and non‐metals. We utilized LLMs to supplement the mechanisms of different microbial synthesis methods for NMs and summarized the top ten associated mechanisms (Figure 3d). Figures S1 and S2 show the process of data cleaning and supplementation by LLMs.

By analyzing the domain knowledge, we obtain the relationship between microorganisms and NMs, and understand the focus of the researches in the domain so far. It is evident that previous research has been empirical, and studies on microbial production of NMs have primarily focused on a limited number of microorganisms and metallic materials. There remains a vast spectrum of species and elements yet to be explored.

### 2.2 Potential Microbes Prediction Model

We propose a novel prediction model for potential microbes based on domain knowledge graph characteristics. The model comprises three key components: (1) a BERT (Bidirectional Encoder Representations from Transformers)‐based module for knowledge entity embeddings, encompassing both microbes and NMs (the structure is as shown in Figure S3); (2) a RGCN (Relational Graph Convolutional Network)‐based module to capture inter‐node dependencies within the domain knowledge graph (the structure is as shown in Figure S4); and (3) a fusion module that integrates textual and relational data outputs. This multi‐faceted approach enhances the model's capacity to comprehend complex interactions and contextual information embedded in the knowledge graph structure (refer to Methods for details).

The model has two main purposes. First, it aims to generate an embedded representation for each node in the knowledge graph. This representation helps capture key information about the nodes. Second, after obtaining these embeddings, the model calculates the similarity between the fusion embeddings. Based on this similarity score, it can determine whether a microbe has the ability to synthesize NMs. For example, the vector of the microbial node is $m_{1}$, and the vector of the nanomaterial node is $m_{2}$. Then, the scoring formula for these microbial and nanomaterial nodes is as follows:

$$\text{score}=\frac{1}{1+∥m_{1}-m_{2}∥}$$

Therefore, we choose ApproxMRRLoss [33] as the training objective, which can be calculated as follows:

$$\text{ApproxMRRLoss}=-\frac{1}{\left|Q\right|}\sum_{i=1}^{\left|Q\right|}log\left(\sum_{j=1}^{|C_{i}|}\frac{\exp(-s_{ij})}{\sum_{k=1}^{|C_{i}|}\exp\left(-s_{ik}\right)}\right)$$

where Q denotes the set of queries. $C_{i}$ denotes the set of candidate answers for the i‐th query. $s_{ij}$ denotes the score given by the model to the j‐th candidate answer for the i‐th query. $\exp(-s_{ij})$ applies the exponential function to the negated score to smooth the reciprocal rank.

We used the Mean Reciprocal Rank (MRR) and HITS@K (Hits at K) to evaluate the effectiveness of the model. The MRR can be formulated as:

(1) $$\text{MRR}=\frac{1}{\left|Q\right|}\sum_{q\in Q}\frac{1}{r_{q}}$$

where |Q| is the total number of queries, $r_{q}$ is the rank position of the first relevant result for query q.

## 3 Evaluation of MicrobeDiscover

### 3.1 Computational Evaluation

For empirical evaluation, we employ a temporal split, using pre‐2019 data for training and post‐2019 data for testing. Performance metrics include Mean Reciprocal (MR), Mean Reciprocal Rank (MRR), and click‐through rates at different ranks (5, 10, and 20). The description of indicator setting can refer to the Metrics of MicrobeDiscover Section in the supplementary materials.

We benchmark MicrobeDiscover against typical representation methods (BERT, GCN, GAT, R‐GCN, etc.) and fusion representation approaches (BERT‐metapath, LSTM‐GCN, BERT‐GCN, etc.). Table 1 demonstrates MicrobeDiscover's superior performance across all metrics, highlighting its efficacy in characterizing microorganisms within the domain.

**TABLE 1:** Experimental results of MR, MRR, HITS@5, HITS@10, and HITS@20 for each model, the arrow indicates the optimization direction of the metric.

Methods	Training Data	MRR↑	HITS@5↑	HITS@10↑	HITS@20↑
metapath2${\mathrm{vec}}^{5}$	Graph	0.0354	0.0833	0.1011	0.3146
GCN^1^	Graph	0.0942±0.0083	0.1693±0.0385	0.3461±0.0509	0.5641±0.0294
GAT^2^	Graph	0.0867±0.0009	0.1538±0.0770	0.3205±0.1155	0.5385±0.0821
RGCN	Graph	0.0772±0.0082	0.1795±0.0484	0.3397±0.0778	0.5961± 0.1071
fastText^5^	Text	0.0573	0.0417	0.1685	0.2083
GRU^3^	Text	0.1196±0.0140	0.1538±0.0192	0.1987 ±0.0110	0.5128 ±0.0618
LSTM^4^	Text	0.0924±0.0044	0.1923±0.0333	0.3526±0.0555	0.7500±0.1541
BERT	Text	**0.0962** ± **0.0190**	0.1730±0.0333	0.3551±0.0624	0.7372±0.0962
LSTM‐GCN	Graph&Text	0.0801 ±0.0087	0.1731 ±0.0577	0.2821±0.0294	0.5705±0.0889
LSTM‐RGCN	Graph&Text	0.0813±0.0158	0.1795±0.1250	0.3077±0.1443	0.6538±0.1154
BERT‐metapath2vec^5^	Graph&Text	0.0772	0.1250	0.2584	0.3596
BERT‐GCN	Graph&Text	0.0744±0.0076	0.1410±0.0111	0.2564±0.0222	0.4167±0.0111
MicrobeDiscover	Graph&Text	0.0930±0.0065	**0.2244** ± **0.0588**	**0.4295** ± **0.0618**	**0.8077** ± **0.0192**

^1^ Graph Convolutional Network.

^2^ Graph Attention Network.

^3^ Gated Recurrent Unit.

^4^ Long Short‐Term Memory.

^5^ No Training Process.

Analysis of the results yields three key findings: (1) MicrobeDiscover outperforms all other evaluated models across multiple metrics, validating the effectiveness of its multi‐modal approach; (2) While HITS@5 proved challenging for all models, MicrobeDiscover demonstrated substantial improvement over the second best competitor; (3) Text‐based features consistently outperformed graph‐based features, highlighting the critical role of semantic information in model performance. These observations underscore the advantages of MicrobeDiscover's integrated architecture in capturing complex domain knowledge.

To verify whether the additional information provided by the LLMs can improve the performance of MicrobeDiscover, we also conducted ablation experiments as shown in Table S2. Moreover, we applied two types of objective in the training of MicrobeDiscover (Table S2). The ablation experimental details can be found in the Supporting Information.

As shown in Figure 4a, the comparative experimental results indicate that MicrobeDiscover performed best. As a first test, we compared our predicted microbes with available literature after 2019, to test the accuracy of prediction results (Figure 4b). The predicted results of single metals and bimetallic NMs were used as examples. For biosynthesis of single metals, we successfully predict *Shewanella oneidensis* MR‐1 [34], *Desulfovibrio ferrophilus* IS5 [35], *Desulfovibrio ferrophilus* ATCC 29577 [35], and *Citrobacter* sp. [36] with Pd, and *Bacillus badius* [37], *Mucor plumbeus* [38], *Vibrio alginolyticus* [39], *Pseudomonas stutzeri* KDP_M2 [40], and *Escherichia coli* ATCC35218 [41] with Au. For biosynthesis of bimetallic AuPd, *Rhodopseudomonas palustris* KRPR01, and *Rhodopseudomonas palustris* KRPR02 [42] are appeared in the predicted results. Other microbes that have not yet been studied for biosynthesis of NMs are considered to be predictions that can be tested in the future, rather than dismissing these instances as spurious.

To verify the impact of the scale of training data on the performance of the model, this paper designs an incremental time series experiment. Specifically, the data after 2019 is fixed as the test set to maintain the stability and fairness of the test data. At the same time, a progressive training set construction strategy is adopted: historical data accumulated year by year, such as data before 2011, before 2012, before 2013, etc., are used to construct the training set respectively, forming nine sets of comparative experiments (from 2011 to 2019) with temporal continuity. Through this design, the relationship between the scale of training data and the model performance indicator is systematically investigated, which is as shown in Figure S5. The experimental results show that as the volume of training data increases, various evaluation indicators exhibit an upward trend. The improvement of the complete training set in terms of the MRR evaluation index compared with the training set before 2011 reaches 80.77%, which proves the continuous optimization of the ranking quality. It can be seen that the accumulation of historical data effectively enriches the representation of entity relationships and enhances the model's ability to capture information.

### 3.2 Potential Microbes Prediction Analysis

MicrobeDiscover was then used to make predictions of potential microbes in NMs synthesis. First, we screened from the annotated microorganisms to find the ones able to produce trimetallic NMs. Trimetallic NMs have been demonstrated with high catalytic performances for some chemical reactions [43, 44] and were difficult to synthesize using traditional approaches. To our knowledge, they have never been reported to be synthesized through microorganisms yet. We normalized the MRR index, in this way a score of 1 indicates it ranks first, but it does not guarantee 100% material production (the detailed convert method in Supporting Information). Taking four trimetallic NMs, AuPdPt, AgPdPt, AuAgPt, and AuAgPd as an example, Figure 5a suggests three top microorganisms of *Shewanella oneidensis* MR‐1, *Bacillus* sp. GP and *Thermus viride* MTCC 5661. As a typical electroactive microbe, *Shewanella oneidensis* MR‐1 has attracted much attention due to its unique extracellular electron transfer ability [45, 46] and outstanding performances in producing metal sulfides, metal oxides, metalloid, single metals and even bimetals [11, 47], but no reports of trimetallic NMs.

Second, we selected microorganism strains that have never been reported in synthesizing NMs to predict their ability of producing Ag, Au, Pd, Pt, AuAg, AuPd, AuPt, AgPd, AgPt, PdPt, AuPdPt, AgPdPt, AuAgPt, and AuAgPd. These strains were from 24 microbial available in China. The results are shown in Figure 5b. Among these microorganisms, *Pantoea agglomerans* ATCC 27155 ranked highest and had good predictive performances in a variety of trimetallic NMs. The details are listed in Table S3.

### 3.3 Statistical Analysis

As shown in Table 1, the mean and standard deviation of test root mean square error on three independent runs are reported (where metapath2vec, BERT‐metapath2vec and fastText are based on semantic similarity calculation, there is no training process, so the results remain consistent across multiple runs). To better assess the reliability of the results, we added 95% confidence intervals (CIs) computed via three independent runs, which are [0.0769, 0.1091] for MRR, [0.0784, 0.3703] for HITS@5, [0.2760, 0.5829] for HITS@10, and [0.7600, 0.8554] for HITS@20. These CIs indicate the stability of our model's performance. We also employed the leave‐one‐genus‐out validation method for evaluation, the results are list in Table S2. The other analysis of MicrobeDiscover can be found in Supporting Information (Figure S6).

## 4 MicrobeDiscover Accelerated Synthesis of NMs

The above results provide a comprehensive analysis of MicrobeDiscover. To assess MicrobeDiscover's prediction capability, we examined its performance by actual materials preparation and online application.

### 4.1 Material Preparation Experiment

First, we conducted NMs synthesis experiments based on the MicrobeDiscover's recommendations to evaluate the reliability of MicrobeDiscover.

*Shewanella oneidensis* MR‐1 was recommended with highest average score on the synthesis of AuPdPt, PdAgPt, PtAuAg, and AuPdAg. Experimental details are in the Supporting Information. From the Scanning Electron Microscope (SEM) images in Figure 6a, abundant of nanoparticles are distributed on the surface of *Shewanella oneidensis* MR‐1 cells. By further looking into the Energy Dispersive Spectrometer (EDS) mapping results, the particles are composed of Au, Pd, and Pt elements uniformly. High‐resolution transmission electron microscopy (HR‐TEM) data show a lattice distance of 0.229 nm, suggesting the possible existence of ternary alloy nanoparticles as reported previously [48].The selected‐area electron diffraction (SAED) pattern in Figure 6b shows four diffraction rings, corresponding to the (111), (200), (220), and (311) planes of a face‐centered cubic structure as indicated by the characteristic diffractions of these individual metals. We conducted STEM‐EDS line scan analysis (Figure S7) to provide information on element distribution at the nanoscale. The results show that the peak positions of Au, Pd, and Pt were consistent, which indicates that the three elements were spatially consistent and provide support for the formation of nanoscale alloy phases. The X‐ray photoelectron spectroscopy (Figure S8) shows that Au existed in metallic state, Pd and Pt mainly existed in oxidized states such as PdO, PtO, or hydroxides. It Indicates that on the surface of alloy nanoparticles, palladium and platinum with higher chemical activity preferentially oxidized to form a surface oxide layer, while inert gold remained in a metallic state. We also analyzed the particle sizes of two repeated synthesis experiments, The average diameter of the nanoparticles were 18.32 nm ± 8.54 nm and 19.91 nm ± 7.12 nm respectively, indicating the reproducibility of the synthesis experiment (Figure S9). Besides AuPdPt, *Shewanella oneidensis* MR‐1 was also found capable of synthesizing other three kinds of trimetallic NMs, although the morphologies were not well controlled in our experiments (Figures S10– S12). Even so, since *Shewanella oneidensis* MR‐1 has never been demonstrated to produce any trimetallic NMs, our experimental results confirmed the reliability of MicrobeDiscover.

Moreover, we also used *Pantoea agglomerans* ATCC 27155 which has not been previously reported to synthesize NMs, to synthesize trimetallic NMs. *Pantoea agglomerans* ATCC 27155 had a high score to synthesize trimetallic NMs among available microorganisms as mentioned above. SEM and EDS mapping in Figure 6c show that there are nanoparticles distributing on the surface of cells with uniform appearence of PtPdAg elements. However, the synthesis of the other three trimetallic materials in Figures S13– S15 were not successful. Even so, no previous reports have shown that *Pantoea agglomerans* ATCC 27155 can synthesize any NMs. The successful synthesis of PtPdAg trimetallic NMs demonstrated MicrobeDiscover's ability to recommend new microorganisms for the synthesis of NMs.

To further validate the accuracy of MicrobeDiscover's prediction results, negative control experiments were conducted. We used MicrobeDiscover to evaluate the potential of currently available microorganisms for producing trimetallic NMs in Figure 5b and Table S3. The predicted results also indicated that among all available microorganisms, *Aspergillus carbonarious* CICC 2215 ranked last in performance score, with an average score of only 0.08. Experimental validation through synthesis experiments in SEM and EDS mapping as demonstrated in the Figures S16– S19 revealed that *Aspergillus carbonarious* CICC 2215 predominantly generated gold NMs, with no obvious formation of trimetallic nanostructures, which was consistent with the predicted result that *Aspergillus carbonarious* CICC 2215 had low possibility to synthesize trimetallic NMs.

### 4.2 Applications of Potential Microbial Prediction

Owing to regional limitations, the predictive and preparative experiments in this study were initially confined to a predefined set of microorganisms. However, the developed MicrobeDiscover framework exhibits robust scalability to accommodate a broader microbial repertoire. To address this constraint and provide comprehensive insights for the research community, we extended our predictive experiments to encompass microorganisms retrieved from the NCBI database.The NCBI database houses vast amounts of phylogenetic tree information, effectively providing models with an extensive “knowledge base.” It is not feasible for scientists to experimentally investigate every microorganism for its ability to synthesize nanomaterials. By integrating diverse microbial strains from across the globe into models through phylogenetic trees, MicrobeDiscover can infer the potential for nanomaterial synthesis by leveraging evolutionary relationships and associated mechanistic information. Furthermore, through the incorporation of NCBI data, MicrobeDiscover can identify evolutionarily conserved features linked to synthesis capabilities. If numerous phylogenetically dispersed and unrelated microorganisms are found to synthesize a specific nanomaterial, MicrobeDiscover can retrospectively infer that an ancient and conserved metabolic pathway or enzyme family is likely driving this synthetic process. This provides valuable clues for uncovering universal synthesis mechanisms. Therefore, we introduced microorganisms from NCBI to expand the predictive ability of MicrobeDiscover. Specifically, starting from the literature‐reported microbial strains with validated synthesis capabilities, we leveraged phylogenetic tree analysis to associate a diverse array of microorganisms, species, and genera from NCBI. Based on this expanded dataset, we systematically predicted the tri‐metallic alloy synthesis potential of these microorganisms.

We introduced 12 558 microorganisms by phylogenetic tree, and predicted their abilities in trimentallic NMs synthesis. First, MicrobeDiscover learned from 12 558 microorganisms and predicted their ability to synthesize nanomaterials at the genus level. Figure 7a presents a box plot illustrating the distribution of predicted potential at the genus level. Genera such as *Sargassum*, *Callipia*, and *Hericium* exhibit relatively high median predicted values, indicating that microbial strains within these genera possess a stronger propensity for trimetallic NM biosynthesis. This indicates that the ability to synthesize trimentallic NMs was not randomly distributed, but rather exhibited a certain degree of aggregation in the microbial phylogenetic tree. It suggests that the synthesis ability of nanomaterials may serve as a heritable microbial functional trait, providing clear evolutionary biology clues for exploring the genetic and metabolic mechanisms behind it. It also guides researchers to prioritize screening microorganisms from these high potential genera, greatly improving the efficiency of discovering efficient synthetic strains. Furthermore, MicrobeDiscover can also make predictions at the species level. Figure 7b demonstrates the predicted trimetallic nanomaterial (NM) biosynthesis potential of individual microbial species, representative taxa with relatively high predicted values include *Neobacillus jeddahensis*, *Callipia waterfrieindii*, and *Condnascusus tortuosus*. The detailed results can be found Table S4. To evaluate the potential performance of different bacterial strains in ternary metal synthesis, we visualized the top 20 prediction strains with the overall performance, as shown in Figure S20. Through the prediction of the model, scientists can systematically conduct validation experiments based on this ranking, thereby concentrating valuable experimental resources on the direction with the highest probability of success. We also employed hierarchical clustering for for analyzing the patterns of more closly related trimetallic NMS and reported microorganisms, which as shown in Figure S21. Cluster analysis preliminarily decoded the association between microorganisms and their synthetic products. This analysis suggests that certain microbiota that were not related to phylogenetics may tend to synthesize similar trimentallic NMs, while certain microbiota that were closely related may synthesize different NMs. This pattern strongly suggests the existence of conserved metabolic networks or gene modules beyond species boundaries that regulate the synthesis of specific NMs. This was not only a reflection of the model's predictive ability, but also helps us to further analyze the biosynthetic mechanisms and even reconstruct these pathways through engineering methods in the future.

Furthermore, we visualized the predicted potentialities and developed a user‐friendly web platform to facilitate rapid retrieval of target microorganisms for researchers in the field of microbial synthesis of nanomaterials. This demo website is accessible via http://catalystdeveloper.sciwiki.cn/m2m. The overview of the system is shown in Figure 8 and Figure S22.

As an AI‐based framework, MicrobeDiscover's core value lies in its ability to break through the limitations of traditional microbial screening methods and achieve more efficient and accurate mining and utilization of microorganisms. It could also potentially break the limitations of microbial biosynthetic ability. The emergence of these new materials will broaden the research field of NMs microbial synthesis.

## 5 Discussion

Sustainable microbial synthesis of NMs holds great promise in energy, catalysis and environment areas. As a NMs synthesis technology, it can reduce chemical consumption and minimize secondary pollution, which is environmentally and suitable for large‐scale production of NMs. In this work, we proposed MicrobeDiscover, an AI‐based framework with domain knowledge fusion to discover potential microbes for microbial synthesis, and applied it to assist the NMs biosynthesis. By utilizing semantic information and association information for representation learning, MicrobeDiscover demonstrated superior performance in discovering potential microorganisms for NMs microbial synthesis, suggesting its potential as an experienced assistant for scientists in the fields of materials, microbiology, energy, and environment.

Using MicrobeDiscover, we predicted and analyzed the microbial potential in synthesizing new materials and conducted synthesis experiments based on the predicted results. Typically in the case of *Shewanella oneidensis* MR‐1 as recommended by MicrobeDiscover, we successfully synthesized AuPdPt in the possible form of the alloy, and AgPdPt, AuAgPt, and AuAgPd as trimetallic NMs, which have never been realized in biosynthesis manner. This extends the capability of *Shewanella oneidensis* MR‐1, and more importantly, validated the effectiveness of MicrobeDiscover.

More microorganisms were efficiently screened from nearly 12 558 microorganisms provided by NCBI for the synthesis of NMs. The discovery of both new materials and new microorganisms will greatly expand the scope of research in this field. Furthermore, MicrobeDiscover may also be expanded into microbial synthesis of other types of materials, such as degradable plastics, polymer materials, etc., promoting the development of new research paradigms.

However, several limitations still persist. One major constraint is the lack of genetic level information, which is important in bioinformatics. The complexity of this type of information is one reason, which needs more effective feature engineering methods, otherwise it will reduce the predictive performance of the model. The second bottleneck is that the construction of benchmark knowledge datasets still requires a certain amount of expert resources. Despite these limitations, this study demonstrates the promising ability of MicrobeDiscover to offer novel and insightful perspectives to assist scientists in biosynthesis of material.

In conclusion, our framework, MicrobeDiscover, is the first ML‐based method for potential microbes discovery in NMs biosynthesis. This work integrates knowledge data from the fields of microbiology and materials based on natural language processing method and large language model, and construct a high‐quality knowledge graph of microbial synthesis of NMs. It bridges massive microbial mechanism, phylogenetic tree, and other microbial field data with chemical material field data such as elements, compound composition and properties. Based on this, we developed a deep learning model that integrates semantic information and graph information, and validated it's effectiveness through various methods, include computational evaluation and synthesis experiments. We also provided an online application for retrieving all results.

MicrobeDiscover can be used in the following scenarios:

- Scientific teams with batch experiment capabilities (or with automatic robots) can directly conduct batch experiments according to the recommended top 20 to obtain rapid preparation results.
- Scientific teams with specific strains can selectively conduct experimental verification on high probability NMs based on the ranking of the predicted results provided by MicrobeDiscover.
- Scientific teams with target NMs can select high probability and obtainable microorganisms for preparation experiments through the sorting of microorganisms.

## Author Contributions

All authors contributed substantively to the work presented in this paper. Conception and Supervision: Y. Du and B. Wang; Data acquisition: Q. Huang and Y. Liu; Data validation: Q. Huang, H. Han, B. Wang, and Y. Gao; Model design and implementation: L. Wang, Y. Liu, Z. Ning, and Y. Du; Model evaluation: L. Wang, Y. Liu, Z. Ning, and Y. Du; Case study: H. Han, Y. Ma, and B. Wang; Technical validation: L. Wang, Y. Liu, Y. Ma, H. W, and J. H; Dataset mining: H. Han, Y. Gao, and B. Wang; Website design and implementation: L. Wang, H. Han, Y. Ma, W. Cui, and Y. Du; Writing and Proof reading: W. Cui, Y. Zhou, H. Han, L. Wang, Y. Gao, B. Wang, and Y. Du.

## funding

Natural Science Foundation of China under Grant Nos. T2322027 and 62442204, the National Key R&D Program of China (2022YFF0712200, 2022YFF0711900), Information Science Database in National Basic Science Data Center under Grant No. NBSDC‐DB‐25, the Young Elite Scientists Sponsorship Program by Beijing Association for Science and Technology under Grant No. BYESS2023410, the Youth Innovation Promotion Association CAS.

## Conflicts of Interest

The authors declare no conflicts of interest.

## Supporting information


**Supporting File**: advs73564‐sup‐0001‐SuppMat.pdf.

## Data Availability

The domain knowledge dataset can be accessed by 10.57760/sciencedb.10875 [28]. The source code of this work is freely available in the GitHub repository https://github.com/kg4sci/MicrobeDiscover. This demo website is accessible via http://catalystdeveloper.sciwiki.cn/m2m.

## Appendix A Knowledge

In this work, we systematically harvest and consolidate knowledge from three principal sources: scholarly literature, domain‐specific databases, and LLMs, utilizing tailored extraction methodologies for each. Specifically, (1) we extract and annotate domain‐specific entities, attributes, and inter‐domain relationships from scientific texts, forming a foundational schema for further knowledge enhancement; (2) from the NCBI portal, we gather comprehensive attributes and phylogenetic data (developmental tree information), facilitating the introduction of novel microbial entities; (3) leveraging LLMs, we enhance microbial attribute databases and refine the data through meticulously crafted prompts. Details on these methodologies are elaborated in the Supporting Information.

### A.1 Knowledge Ontology Design

For the construction of the domain knowledge datasets, we design an ontology. In the proposed ontology, we provided eight entity labels including microorganism, mechanism, synthesis method, location, size, shape, element, and 14 entity attributes, including habitat, function, metal resistance, precursor, pH, temperature, rotate speed, application, catalysis, biochemistry, antibacterial agent, electronics, energy, and other information. The specific ontology content is shown in the Table S1.

### A.2 Entity Annotation

The full papers were operated differently according to the way the different corpora were constructed. In order to obtain the data needed for database construction, articles were retrieved from Web of Science Core Collection database to find the DOIs of the scientific literature, and only articles published in English were included, as shown in Figure A1. First, the metadata of more than 22 121 articles were exported from the Web of Science website using the keywords “biosynthesis” or “microbial synthesis” or “bacterial synthesis” and “nano*” as the subject index, which included the article title, article DOIs, article abstracts, etc. Then 13 876 articles were obtained by querying “plant” in the topic of each article and then deleting them. 4981 articles were obtained by querying “microorganism” or “microbe” or “bacteria” in the topic of each article. A total of 2914 articles were obtained by querying “engineering” in the topic of each article and then removing it. Finally, 536 articles related to the synthesis of nanomaterials by microorganisms were obtained by further manual screening by experts.

Figure A2 provides the flowchart of the annotation process in this paper, and we invited five postgraduates with an average experience of at least 3 years in experimental synthesis from Institute of Urban Environment, Chinese Academy of Sciences and onw postdoc from National Center for Nanoscience and Technology to do the work after the annotation tool training. In the process of building the knowledge base, we fully leverage the advantages of both parties in cooperation. First, domain experts annotated the domain knowledge contained in scientific and technological literature with the provided annotation tools, and then hand it over to computer professionals for statistics and visualization. Second, refer to the analysis results, domain experts revise the annotated knowledge, and computer professionals analyze and evaluate it again. After being tested by domain experts, The standard domain knowledge base obtained.

### A.3 LLMs Enhanced Data Science

Publications serve as a pivotal resource for scholars, particularly within multidisciplinary studies where insights are primarily derived from scholarly articles, exemplified by the domain of microbial NMs synthesis. Concurrently, specialized data can often be further scrutinized through domain‐specific open datasets. For example, in the field of microbiology, NCBI provides access to its biomedical and genomic information, which is more comprehensive and precise. Furthermore, the advent of Large Language Models (LLMs) has enabled the utilization of their robust search and summarization functions to augment the scope of domain‐specific knowledge.

1. **Material Standardization** In the standardization of material category entities, we performed the following processing using large language models: (1) Corrected incorrectly labeled materials (e.g., Agg −> Ag); (2) Aligned different descriptions of the same material (e.g., Silver −> Ag, ag −> Ag); (3) Identified constituent elements of alloy and compound materials (e.g., zinc oxide −> Zn + O, Calcium carbonate −> Ca, C, O).
2. **Microorganism Standardization** The standardization of microorganisms using LLMs involves three core tasks: (1) Complete Abbreviations. Expand abbreviated microbial names into their full standard forms. For example, the abbreviation “A. Flavus” is completed to the full name *Aspergillus Flavus*. (2) Correct Naming Irregularities. Standardize the formatting and writing of microbial names. For instance, the non‐standard lowercase form “aspergillus Flavus” is corrected to the “*Aspergillus Flavus*”.
3. **Synthesis Mechanism Supplementation** We also add relevant functional mechanism details to the microbial entry by LLMs. For example, the mechanism “Hydrogenase” is supplemented for *Aspergillus Flavus*.

## Appendix B Method

### B.1 Semantic Modular

MicrobeDiscover uses BERT to extract the semantic information since it has strong semantic representation advantages. The BERT model uses a multi‐layer bidirectional Transformer encoder structure [49]. The Transformer model is a new architecture of the text sequence network, based on the self‐attention mechanism. It mainly adjusts the weight coefficient matrix to obtain the characterization of the word through the degree of relevance between the words in the same sentence. Given a sample $S=attr_{1},\text{…},attr_{n}$, MicrobeDiscover uses BPE tokenizer to turn the source sequence into corresponding wordpiece sequence as:

(B1)
$$S=W_{attr_{1}}^{\#1},\text{…},W_{attr_{1}}^{\#k_{1}},\text{…},W_{attr_{n}}^{\#1},\text{…},W_{attr_{n}}^{\#k_{n}}$$

where $W_{i}^{\#j}$ represents the j‐th sub‐word of word, and means the number of sub‐words in word. BERT model consist of several identical layers of Transformer encoder, including the multi‐head self‐attention module, the normalized layer and position‐wise feed forward networks. The input representation of one token can be calculated by summing the corresponding token, segment and position embedding, and the overall embedding for the i‐th word and the output representation of sequence can be obtained as:

(B2)
$$e^{i}=e_{tok}^{i}+e_{seg}^{i}+e_{pos}^{i}$$

(B3)
$$E=e^{1},e^{2},\text{…},e^{M}$$

(B4)
$$E_{semantic}=H^{seq}=BERT\left(E\right)$$

The BERT in this paper consists of 12 Transformer encoder layers, 12 attention heads per layer, and a hidden size of 768, totaling 110 million parameters. This model is pretrained on the BookCorpus (about 800 million tokens) and English Wikipedia (about 2.5 billion tokens excluding lists, tables and headers), and the two corpora together consist of approximately 3.3 billion tokens.

### B.2 Graph Modular

MicrobeDiscover uses RGCN [50] as the base module in the Graph Modular. RGCN is a network model developed specifically for handling highly multi relational data features in real‐world knowledge bases, which has become been widely adopted for combining knowledge graphs with machine learning applications.

For the problem of node isomorphism and edge heterogeneity, RGCN provides a good and universal approach to dealing with relatively simple heterogeneous graphs, which is divide and conquer. The formula is as follows:

(B5)
$$E_{graph}=h_{i}^{\left(l+1\right)}=\sigma \left(\sum_{r\in R}\sum_{j\in N_{i}^{r}}\frac{1}{c_{i,r}}W_{r}^{\left(l\right)}h_{j}^{\left(l\right)}+W_{0}^{\left(l\right)}h_{i}^{\left(l\right)}\right)$$

Here, $N_{i}^{r}$ represents the set of neighbor nodes connected to node v through relationship r; $W_{r}^{\left(l\right)}$ is the transformation matrix of relationship r at the l‐th layer; $h_{u}^{\left(l\right)}$ is the feature vector of node u in the l‐th layer; $W_{0}^{\left(l\right)}$ is the transformation matrix of the node's own information; $c_{i}^{\left(l\right)}$ is a normalization constant used to adjust the contribution of different relationship types to the node representation update; σ is a non‐linear activation function, and the ReLU function is used in MicrobeDiscover.

After training, we take the latent variables as the corresponding output node embedding. Thus the overall embedding of input, is:

(B6)
$$O=W_{output}\left(E_{semantic}+E_{graph}\right)$$
