# Supplementary material for: MicrobeDiscover: A Knowledge Graph–Enabled AI Framework for Identifying Microbes for Inorganic Nanomaterial Biosynthesis
Source: Adv Sci (Weinh). 2026 Jan 25;13(20):e20587. doi: 10.1002/advs.202520587 (PMC13067785; doi:10.1002/advs.202520587)
Supplement: Supplementary file 1 — Supporting File: advs73564‐sup‐0001‐SuppMat.pdf. [file ADVS-13-e20587-s001.pdf]

WILEY

Allergy EUROPEAN JOURNAL OF ALLERGY AND CLINICAL IMMUNOLOGY EAACI

SUPPORT INFORMATION OPEN ACCESS

MicrobeDiscover: AI-accelerated Discovery of Potential Microbes for Synthesis of Inorganic Nanomaterials

Ludi Wang[1,2] | Hexing Han[4] | Yufeng Liu[1] | Zhiyuan Ning[1] | Yujie Ma[1,2] | Haidan Wang[1,2] | Jinling Xu[3] | Qiansheng Huang[5] | Wenjuan Cui[1,2] | Yuanchun Zhou[1,2,3] | Yang Gao[4] | Bin Wang*[6] | Yi Du*[1,2,3]

[1]Computer Network Information Center, Chinese Academy of Sciences, Beijing, 100083,China | [2]University of Chinese Academy of Sciences, Beijing, 100049, China | [3]Hangzhou Institute for Advanced Study, UCAS, Hangzhou, 310000, China | [4]CAS Key Laboratory of Nanosystem and Hierarchical Fabrication, National Center for Nanoscience and Technology (NCNST), Beijing, 100190, China [5]Institute of Urban Environment (IUE), Chinese Academy of Sciences, Xiamen, 361000, China | [6]Xizang University, Lhasa 850000, China | [7]Key Laboratory of Rare Earths, Jiangxi Institute of Rare Earths, Ganjiang Innovation Academy, Chinese Academy of Sciences, Ganjiang 341119, China |

Received: Revised: Accepted:

Scientific Literature and Its Annotation

Scientific literature is the main vehicle for documenting scientific discoveries and their knowledge, and it contains a great deal of knowledge about microorganism synthetics. We mainly focus on the knowledge related to microorganism synthesized material. For the construction of the domain knowledge datasets, we design an ontology. In the proposed ontology, we provided 8 entity labels including microorganism, mechanism, synthesis method, location, size, shape, element, and 14 entity attribute, including habitat, function, metal resistance, precursor, pH, temperature, rotate speed, application, catalysis, biochemistry, antibacterial agent, electronics, energy, and other information. The specific entity category is shown in Table S1

TABLE S1 | Entity category and the corresponding descriptions.

Entity Category	Description	Count
Microorganism	The microorganism used for the synthesis of nanomaterial.	167
Mechanism	The mechanism of synthesis of nanomaterial by the microorganism, i.e. nitrate reductase.	279
Material	The nanomaterial synthesized by microorganisms, i.e. CdS.	86
Synthesis method	The synthesis method of the nanomaterial by microorganism.	477
Location	The location of the nanomaterial in relation to the microorganism, including intracellular and extracellular, etc.	132
Size	The particle size of nanomaterial.	435
Shape	The shape of nanomaterial.	101
Element	Types of elements contained in the material.	48

LLMs enhanced data science

Publications serve as a pivotal resource for scholars, particularly within multidisciplinary studies where insights are primarily derived from scholarly articles, exemplified by the domain of microbial NMs synthesis. Concurrently, specialized

data can often be further scrutinized through domain-specific open datasets. For example, in the field of microbiology, NCBI provids access to its biomedical and genomic information, which is more comprehensive and precise.
Furthermore, the advent of Large Language Models (LLMs) has enabled the utilization of their robust search and summarization functions to augment the scope of domain-specific knowledge. In this work, we systematically harvest and consolidate knowledge from three principal sources: scholarly literature, domain-specific databases, and LLMs, utilizing tailored extraction methodologies for each. Specifically, 1) we extract and annotate domain-specific entities, attributes, and inter-domain relationships from scientific texts, forming a foundational schema for further knowledge enhancement; 2) from the NCBI portal, we gather comprehensive attributes and phylogenetic data (developmental tree information), facilitating the introduction of novel microbial entities; 3) leveraging LLMs, we enhance microbial attribute databases and refine the data through meticulously crafted prompts. Details on these methodologies are elaborated in the Supplementary materials.
After the construction of annotation labels, we use AutoDive[1], an easy-to-use on-site annotation tool with graphical user interface, to annotate the standard knowledge base. AutoDive provides the label interface in the form of PDF, which can ensure the layout of the original documents that can keep the original habit of reading literature. This tool does not require local installation on the curators side and can be used through a web-browser to make the annotation process as easy and fast as possible.
By counting the year of publication of the relevant articles, we fothere was a general increase trend in the quantity of articles on the microbial synthesis of nanomaterials during the period of 2001-2021. It shows that the research is still a concern for researchers.The field of nanomaterial synthesis research has gradually expanded in scope, moving from focusing on single-metal synthesis to include a variety of material kinds.

NCBI DATABASES

The National Center for Biotechnology Information (NCBI) serves as a pivotal resource in biological research, offering a comprehensive repository of genomic data and associated information. In this study, we use the open API provided by NCBI to obtain the microbial mechanism and phylogenetic trees related with annotated microorganisms. In addition to supplementing information on microbial properties, we also use phylogenetic trees to associate different microorganisms, which can be seen as an association relationship of the domain knowledge graph (detailed introduction will be provided in the following section).
The primary attributes extracted from NCBI for our study include: Taxonomic Classification: This involves the hierarchical classification of microorganisms from domain down to species level. Genomic Information: Includes genome sequences, gene annotations, and functional information related to specific genes. Phenotypic Information: Covers various traits and characteristics of microorganisms, such as growth conditions and metabolic capabilities. The extraction process involves querying NCBI databases using tools such as Entrez Programming Utilities (E-utilities), which provide programmatic access to NCBI's resources. The retrieved data is then parsed and used to populate our knowledge graph with relevant attributes.
Based on the aforementioned data results, the rank attributes of all microbial entities comprise strain, specifications, and genus. Drawing from biological knowledge, We have introduced the lineage association relationship:genus-species-strain. If a microorganism's rank attribute value is a strain, the aim is to identify other strains beneath its superior species within the lineage and establish a strain-species-strain relationship. Similarly, if the rank attribute value of a microorganism is species, the aim is to identify other species both above and below it in the lineage and establish a species-genus-species relationship. Subsequently, a comprehensive lineage association relationship was integrated into the standard domain knowledge base.
In the synthesis of nanomaterials by microorganisms, an understanding of the underlying mechanisms is particularly critical. These mechanisms are not straightforward chemical processes; rather, they entail a sequence of intricate biochemical events that are primarily triggered and controlled by extracellular enzymes, redox-active chemicals, and intracellular enzymes or non-enzymatic proteins 19. These enzymes, non-enzymatic proteins, and redox-active substances often act as catalysts and are engaged in crucial processes in the creation of nanomaterials. The presence or absence of corresponding biomolecules in microorganisms often determines whether nanomaterials can be synthesized. Therefore, in the process of annotating the relevant literature, we paid special attention to the specific role of these biomolecules in the synthesis mechanism and extracted the key enzymes or proteins involved.

1 | Data Processing through Interaction with LLMs

After the above two steps of data acquisition, two issues persist: (1) annotation errors; (2) missing mechanisms in many microorganism entities. LLMs, a cutting-edge technology, process textual data to train models that grasp syntax, semantics, and contextual relationships, enabling the capture of intricate linguistic features. They can produce coherent, grammatically accurate text and sometimes exhibit comprehension of complex contexts. Two of the important capabilities are: 1) storing extensive knowledge; 2) performing tasks interactively with humans. We will leverage these LLM capabilities to address the existing data issues.

1.1 | Data Cleaning

Our current data exhibits several deficiencies and errors, including: 1) missing elements in the data materials, 2) irregularities in the naming of microorganisms and materials, and 3) inconsistencies in material names. Manually programming and cleaning this data would be highly labor-intensive. To address this, we use the ChatGPT dialog for data processing, as depicted in Fig. S1. We clean 612 data points in 7 hours, leaving only 70 uncleaned (failure rate:11.4%).

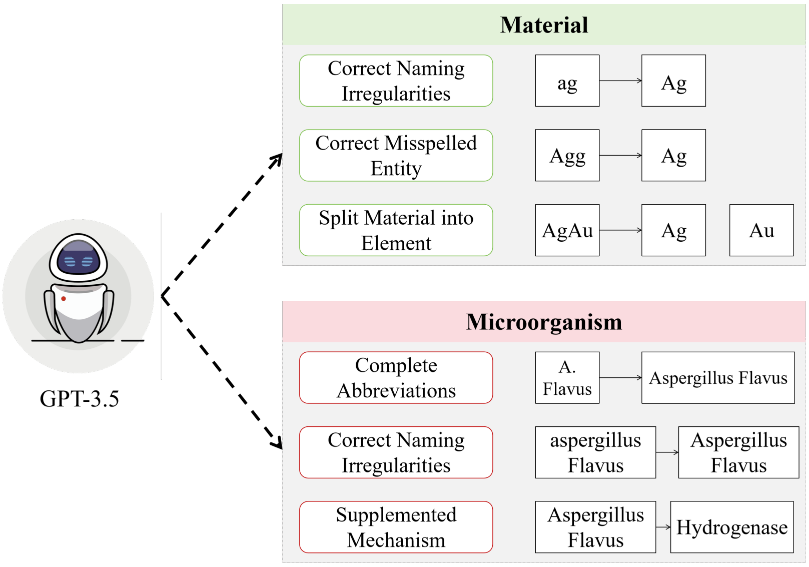

FIGURE S1 | Overview of data cleaning by LLMs.

1.2 | Data Supplementation

After cleaning and duplication, a total of 414 microorganism nodes are included, of which 287 are missing mechanism attribute, but the cost of utilizing an expert to manually perform the mechanism supplementation is huge. LLMs contains a vast amount of knowledge, and we prompt to elicit the knowledge about the mechanism.

To supplement the missing mechanistic data, we employed the GPT-3.5 Large Language Model (LLM). These advanced models can process and generate detailed textual information, making them highly effective for extracting complex data from scientific literature and other textual sources [2]. This approach has already been validated in the field of microbiology for knowledge annotation.The prompts used to query the LLMs were designed to extract specific mechanistic details from textual data. We supplemented 357 microbial mechanism data using a large model, Taking Clostridium thermoaceticum ATCC 3907 as an example, its prompt is shown in the Fig.S2.

In the preliminary dataset annotation process, a total of 1068 entries were initially identified. However, upon closer examination, 456 entries were deemed "not relevant" to our knowledge graph construction methodology and were subsequently excluded from further analysis. Additionally, a significant portion of the dataset lacked annotations regarding the mechanisms of microbial interactions, posing a challenge to the completeness of our knowledge graph. To address this gap, we employed Large Language Models (LLMs) to supplement the missing mechanism data.Furthermore, inconsistencies in data representation, such as variations in material nomenclature (e.g., "Ag" vs. "silver"), were observed within the dataset.

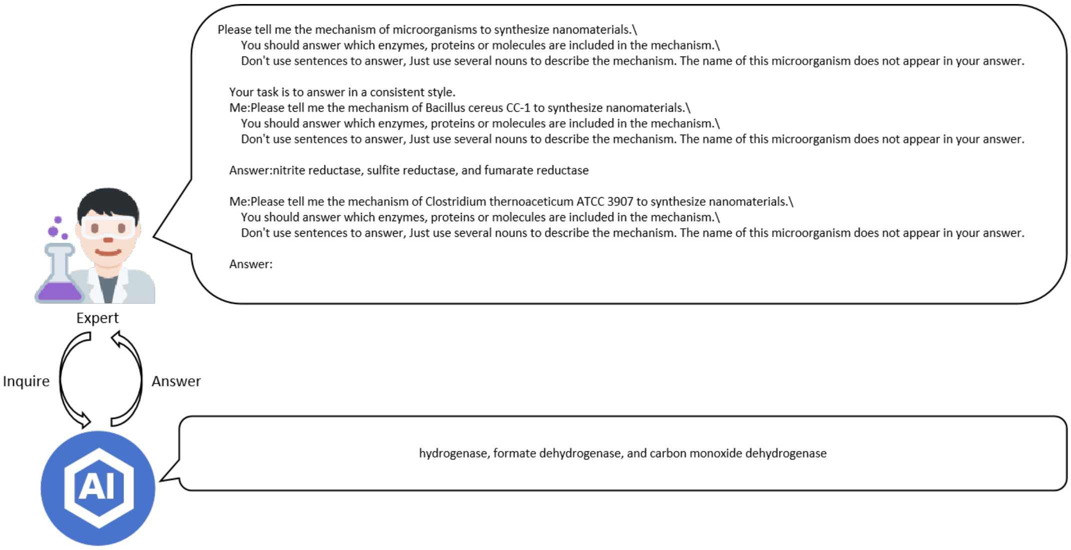

FIGURE S2 | The prompt of data supplementation by LLMs

To mitigate these inconsistencies, we leveraged advanced computational models to standardize the naming conventions. This process involved the utilization of large-scale language models, which enabled automated data normalization procedures. Subsequently, to ensure the accuracy and reliability of the standardized data, a comprehensive manual verification process was conducted.

SEMANTIC MODULAR

MicrobeDiscover uses BERT to extract the semantic information since it has strong semantic representation advantages. As shown in Fig.S3, BERT input representation is spliced by token embeddings, segment embeddings and position embeddings. It is able to clearly represent a single text sentence or a pair of text sentences in a tag sequence. The BERT pre-training language model also captures word-level and sentence-level representations through two tasks, masked language model and next sentence prediction, and conducts joint training. The masked language model is to train a deep bidirectional language representation vector model, and then predict the masked words by randomly masking certain words in the sentence. The masked language model can predict the masked words from any direction. The next sentence prediction is to train a model that understands the relationship between sentences.
The BERT model uses a multi-layer bidirectional Transformer encoder structure[3]. The Transformer model is a new architecture of the text sequence network, based on the self-attention mechanism. It mainly adjusts the weight coefficient matrix to obtain the characterization of the word through the degree of relevance between the words in the same sentence. Given a sample $S = attr_1, ..., attr_n$, MicrobeDiscover uses BPE tokenizer to turn the source sequence into corresponding wordpiece sequence as:

$$S = W^{\#1}_{attr_1}, ..., W^{\#k_1}_{attr_1}, ..., W^{\#1}_{attr_n}, ..., W^{\#k_n}_{attr_n} \quad (1)$$

Where $W_i^{\#j}$ represents the j-th sub-word of word , and means the number of sub-words in word. BERT model consist of several identical layers of Transformer encoder, including the multi-head self-attention module, the normalized layer and position-wise feed forward networks. The input representation of one token can be calculated by summing the corresponding token, segment and position embedding, and the overall embedding for the i-th word and the output representation of sequence can be obtained as:

$$e^i = e^i_{tok} + e^i_{seg} + e^i_{pos} \quad (2)$$

$$E = e^1, e^2, ..., e^M \quad (3)$$

$$E_{semantic} = H^{seq} = BERT(E) \quad (4)$$

GRAPH MODULAR

MicrobeDiscover uses RGCN[4] as the base module in the Graph Modular. RGCN is a network model developed specifically for handling highly multi relational data features in real-world knowledge bases, which has become been widely

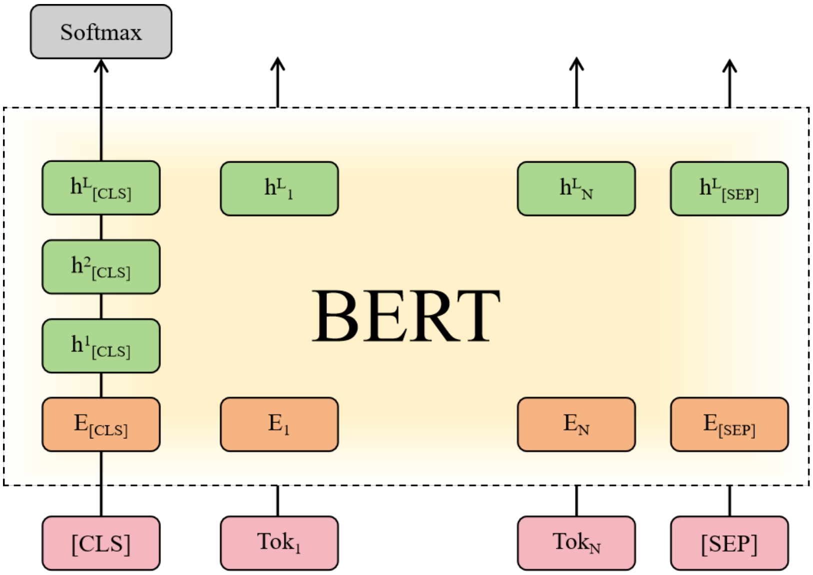

FIGURE S3 | The structure of BERT.

adopted for combining knowledge graphs with machine learning applications. As shown in Fig.S4, RGCN[4] is a network model developed specifically for handling highly multi relational data features in real-world knowledge bases, which has become been widely adopted for combining knowledge graphs with machine learning applications. For the problem of node isomorphism and edge heterogeneity, RGCN provides a good and universal approach to dealing with relatively simple heterogeneous graphs, which is divide and conquer.

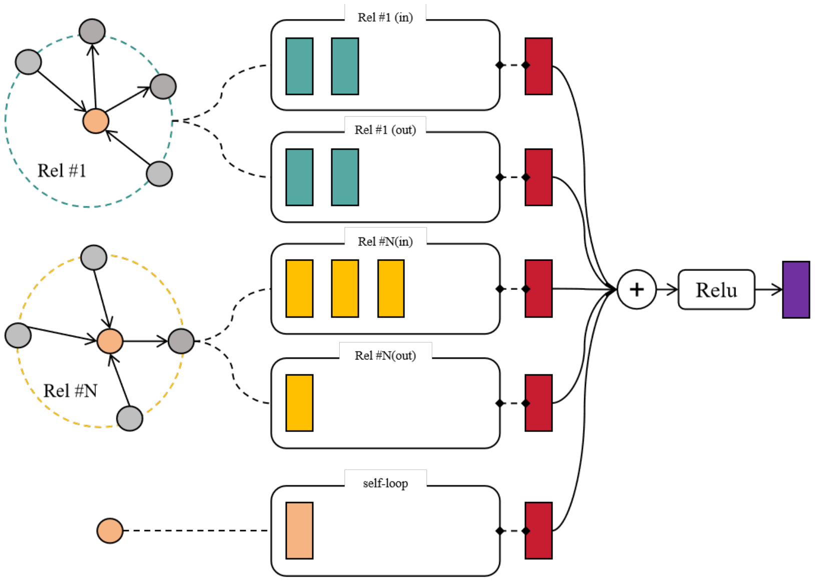

FIGURE S4 | The structure of RGCN.

For the problem of node isomorphism and edge heterogeneity, RGCN provides a good and universal approach to dealing with relatively simple heterogeneous graphs, which is divide and conquer. The formula is as follows:

$$E_{graph} = h_i^{(l+1)} = \sigma(\sum_{r \in R} \sum_{j \in N_i^r} \frac{1}{c_{i,r}} W_r^{(l)} h_j^{(l)} + W_0^{(l)} h_i^{(l)}) \tag{5}$$

After training, we take the latent variables as the corresponding output node embedding. Thus the overall embedding of input, is:

$$O = W_{output}(E_{semantic} + E_{graph}) \tag{6}$$

EVALUATION OF MICROBEDISCOVER

Baseline

metapath2vec

Metapath2vec[5] is a powerful representation learning model designed specifically for heterogeneous information networks (HINs), which contain multiple types of nodes and edges. Introduced by Dong et al. in 2017, metapath2vec leverages metapaths—composite relations connecting different types of nodes—to capture rich semantic relationships and generate meaningful vector representations of nodes. This approach extends traditional homogeneous network embedding techniques, such as DeepWalk and node2vec, to more complex and informative heterogeneous networks. The core innovation of metapath2vec lies in its use of metapaths to guide random walks through the network, ensuring that the generated walks reflect meaningful sequences of node types and relationships. By following metapaths, the model captures higher-order semantic information that is often crucial for tasks like classification, clustering, and recommendation in heterogeneous networks. The metapath-guided random walks produce sequences of nodes that are then used to train a skip-gram model, learning low-dimensional embeddings that preserve both structural and semantic properties of the original network.

GCN

Graph Convolutional Networks (GCNs)[6] are a specialized class of neural networks designed to work with graph-structured data, effectively capturing the relational information inherent in graphs. This is achieved by aggregating feature information from a node's neighbors, using a localized filter defined over the graph's structure. The propagation rule of GCNs, which involves the adjacency matrix and degree matrix of the graph, ensures that the features are appropriately scaled and aggregated, maintaining the structural integrity of the graph.GCNs have demonstrated significant success in various applications, including node classification, link prediction, and graph classification. They have been particularly useful in fields like social network analysis, recommender systems, and molecular biology, where the underlying data naturally forms a graph. Despite their advantages, GCNs face challenges such as computational complexity and the over-smoothing problem, where node representations become indistinguishable with increasing layers.

GAT

Graph Attention Networks (GATs)[7] represent an advancement in the realm of graph-based neural networks, addressing some limitations inherent in Graph Convolutional Networks (GCNs). Proposed by Veličković et al. in 2017, GATs introduce the attention mechanism to graph neural networks, allowing for the assignment of different importance weights to different nodes in a neighborhood when aggregating features. This attention mechanism enhances the model's ability to capture the varying significance of neighboring nodes, thereby improving the expressiveness and performance of graph representations.The key innovation in GATs lies in the use of self-attention to compute attention coefficients for each pair of neighboring nodes. These coefficients determine the weight of each neighbor's contribution to the target node's feature update.

GATs offer several improvements over GCNs. Firstly, GATs remove the requirement for pre-computed graph structure information, such as the normalized adjacency matrix, by learning the attention coefficients directly from the data. This makes GATs more flexible and adaptive to different graph structures. Secondly, the attention mechanism allows GATs to focus on more relevant neighbors, thereby potentially enhancing the learning of node representations in heterogeneous and complex graphs. This dynamic weighting of neighbors addresses the limitation of uniform neighbor aggregation in GCNs.

GRU

The Gated Recurrent Unit (GRU)[8] is a variant of recurrent neural networks (RNNs) designed to handle sequential data with improved efficiency and performance. Introduced by Cho et al. in 2014, GRUs address the shortcomings of traditional

RNNs, such as the vanishing and exploding gradient problems, by incorporating gating mechanisms that regulate the flow of information through the network. This makes GRUs particularly effective for tasks involving time-series data, language modeling, and machine translation.The architecture of GRUs is simpler than that of Long Short-Term Memory (LSTM) networks, another popular type of RNN. GRUs combine the functionalities of the forget and input gates into a single update gate and merge the cell state and hidden state into one. This streamlined design reduces the number of parameters and computations required, making GRUs more computationally efficient and faster to train compared to LSTMs.

LSTM

Long Short-Term Memory (LSTM)[9] networks are a specialized type of recurrent neural network (RNN) designed to address the limitations of traditional RNNs, particularly the issues of vanishing and exploding gradients. Introduced by Hochreiter and Schmidhuber in 1997, LSTMs are capable of learning long-term dependencies in sequential data, making them highly effective for tasks involving time-series data, natural language processing, and other sequence-based applications. The architecture of LSTMs includes memory cells and gating mechanisms that control the flow of information, enabling the network to maintain and update a cell state over long sequences. The key components of an LSTM are the input gate, forget gate, and output gate. The input gate regulates the addition of new information to the cell state, the forget gate controls the removal of irrelevant information, and the output gate determines the portion of the cell state to be used as the output. This structure allows LSTMs to preserve important information over many time steps while discarding unnecessary data.

Hyper-parameters Set

We trained the model using the Adam optimizer, and use the same key hyper-parameters for all models. Detailed hyper-parameter settings for each baseline are provided in Table S2.

TABLE S2 | Hyper-parameter settings.

Parameter	Value
max epochs	50
early stop	4
learning rate	3e-3
weight decay	1e-7
adam parameter(β_1)	0.9
adam parameter(β_2)	0.999
Training Environment	
Operating system	Ubuntu Linux release 18.04
CPU	Intel(R) Xeon(R) Gold 6130
GPU	2 Nvidia A100

Metrics

We utilized the Mean Reciprocal Rank (MRR) and HITS@K (Hits at K) to comprehensively evaluate the effectiveness of our model. The MRR is a well-established metric in information retrieval, providing an average of the reciprocal ranks of results for a set of queries. It is particularly useful for scenarios where the position of the first relevant result is of primary importance. By calculating the MRR, we can quantify how well our model ranks relevant items higher in the results list, thus reflecting its efficiency in providing pertinent information promptly.The MRR can be formulated as:

$$\mathrm{MRR} = \frac{1}{|\Omega|} \sum_{q \in \Omega} \frac{1}{r_q} \tag{7}$$

Where $|\Omega|$ is the total number of queries, r_q is the rank position of the first relevant result for query q.
HITS@K (Hits at K) is a metric used to evaluate the performance of recommendation systems or information retrieval systems. It measures the proportion of queries for which the correct result is found within the top K results. The formula for HITS@K is defined as follows:

$$\text{HITS@K} = \frac{\text{Number of relevant items in top K results}}{\text{Total number of relevant items}}$$

In the context where there is only one relevant item per query, the formula can be simplified as:

$$\text{HITS@K} = \begin{cases} 1 & \text{if relevant item is in top K results} \\ 0 & \text{otherwise} \end{cases}$$

For different values of K, we can define specific metrics:
HITS@5: The proportion of queries for which the correct result is found within the top 5 results.
HITS@10: The proportion of queries for which the correct result is found within the top 10 results.
HITS@20: The proportion of queries for which the correct result is found within the top 20 results.
The original MRR indicators are difficult to distinguish potential microorganisms, so we aligned them and performed scale normalization:

$$SCORE_i = \frac{MRR_i - \max MRR}{\max MRR - \min MRR}(1 - 2\epsilon) + \epsilon \tag{8}$$

where ϵ is e^{-10}.

1.3 | Ablation Study

The input data of MicrobeDiscover integrates both graph and text data characteristic: 1) the graph component outlines the intricate network of relationships among various entities, including microorganisms, materials, elements, synthesis methods, and lineages. This graph-based representation captures the complex interdependencies and interactions inherent in the synthesis processes, providing a rich contextual framework for understanding how different entities are interrelated; 2) the textual component complements this by incorporating detailed descriptions thereby enriching the knowledge graph with semantic depth and contextual nuances. By integrating these two components, our knowledge graph not only maps the structural relationships but also embeds critical textual information, enabling a more holistic understanding of the data. The results are shown in Table S3.
Besides, LLMs provides additional mechanism information for model training, which may bring new insights. Therefore, we also conduct an ablation experiment by removing the information proviede by LLMs, to evaluate the effective of it. The choice of loss function is also one of the important factors affecting the performance of the model. In this study, we compare the two type of loss function: Binary CrossEntropy and ApproxMRRLoss[10].

TABLE S3 | Experimental results on whether or not to use mechanism properties in microorganism

	MRR	HITS@5	HITS@10	HITS@20
**Only Graph Modular**	0.1351	0.1573	0.3708	0.4944
**Only Semantic Modular**	0.1219	0.2247	0.3034	0.4831
**MicrobeDiscover w/o LLM mech**[1]	0.1506	0.2697	0.4494	0.7191
**MicrobeDiscover w/o material property relation**	0.0980	0.2308	0.3462	0.6154
**MicrobeDiscover w/o lineage relation**	0.0976	0.3077	0.4808	0.6731
**MicrobeDiscover w/ BCE Loss**[2]	0.1263	0.2022	0.3708	0.6180
**MicrobeDiscover w/ Approx. MRR Loss**[3]	0.0928	0.1170	0.5383	0.8621
**MicrobeDiscover w/ leave-one-genus-out validation**	0.2143	0.4007	0.6810	0.9876

[1] Exclude information revised or supplemented by LLMs.
[2] Binary CrossEntropy Loss is used of model training, instead of ApproxMRRLoss.
[3] Using original Approx. MRR Loss

From the results, we observe that the combination of graph-info and semantic-info boost the performance, and the inclusion of mechanism attributes allows the model to leverage domain-specific knowledge, which is crucial for accurately characterizing microbial entities. Experimental results indicate that all features, techniques, and modules are effective for the proposed method to achieve performance gains. In the leave-one-genus-out validation, three genera-*Bacillus*, *Pseudomonas*, and *Shewanella*-were selected for model validation, as each has more than 20 positive samples in the dataset (27, 24, and 34 respectively). Notably, compared with time-based data splitting, this validation method yields a substantial improvement in model accuracy due to the reduced size of the test set.

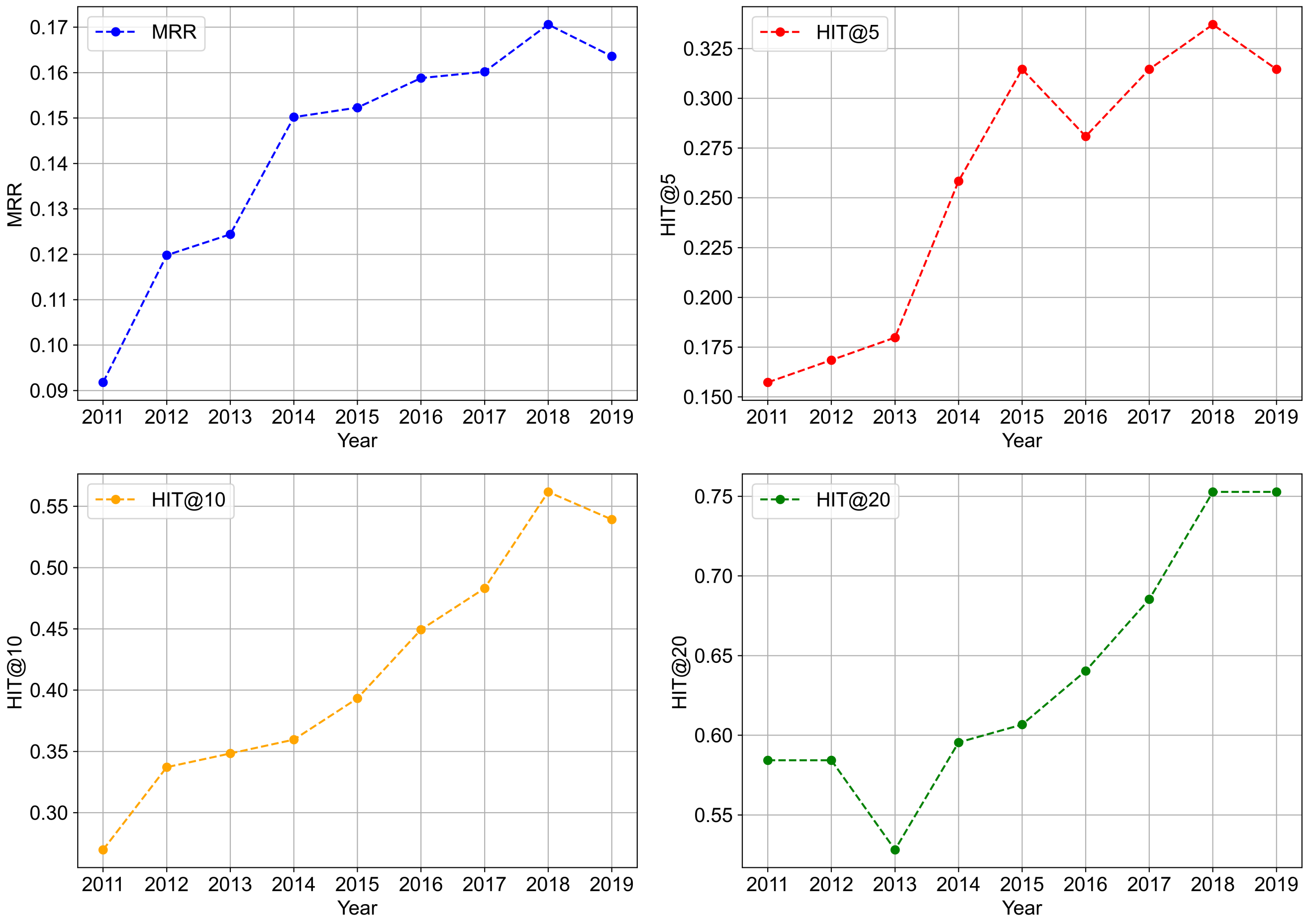

FIGURE S5 | The changing trend of the model's performance after training with training sets divided based on different years.

NEW MICROBIAL DISCOVERIES FOR SYNTHESIZING NMS

To further validate the model, we supplement AUPRC, calibration curves, and uncertainty estimates. To calculate AUPRC, plot calibration curves, and quantify uncertainty estimates, we first converted the ranking recommendation task into a binary classification task, and the continuous relevance scores output by the model were treated as predicted probabilities, based on which AUPRC was calculated; the actual positive rates of samples in different score intervals were statistically grouped to plot calibration curves. We use Monte Carlo Dropout method to predict each positive sample 20 times repeatedly and calculate the score variance as the uncertainty estimate, and the average result was 0.023 which reflect the predictive trustworthiness of the model.
However, as shown in Fig.S6, these metrics performed unsatisfied in this scenario, with the core reasons as follows:
(1) Core cause of poor AUPRC performance: The core of the ranking recommendation task is to learn the relative ranking relationship between samples, rather than absolute judgment in binary classification. After simply labeling such samples as negative, the distribution of positive and negative samples is extremely imbalanced, resulting in an extremely low AUPRC value (only 0.12). This value only reflects one-sided results after binary classification and cannot reflect the core ability of the model to rank highly relevant samples at the top;
(2)Calibration curves lack effective interpretability: The core of calibration curves is to verify the "matching degree between predicted probabilities and actual positive rates". However, the relevance scores output by the ranking model are designed to optimize Top-K ranking (aiming to improve Hits@k), rather than strict probability distributions. When scores are forcibly regarded as probabilities to plot calibration curves, the actual positive rates in different score intervals fluctuate irregularly, and the curves deviate significantly from the ideal calibration line, failing to reflect the calibration characteristics of the model.

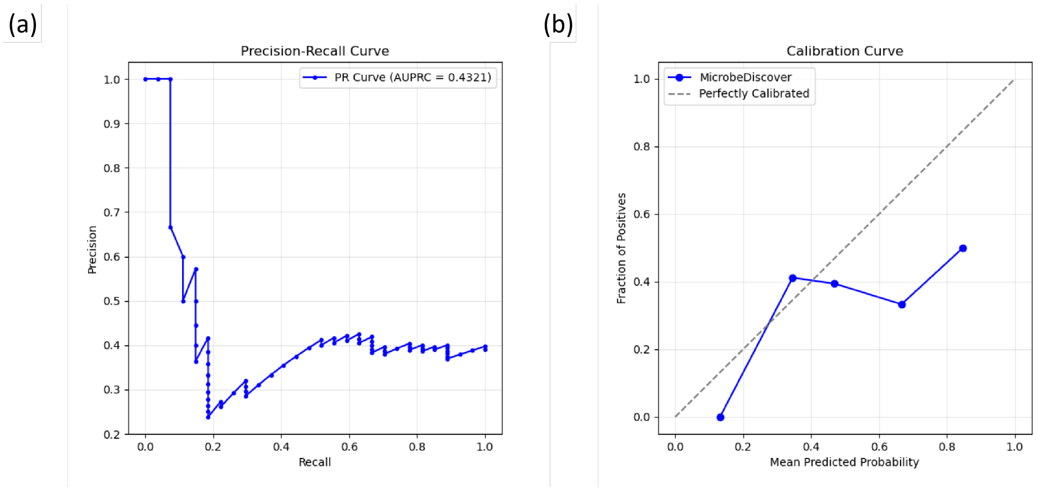

FIGURE S6 | (a) The Precision-Recall Curve of MicrobeDiscover; (b) the Calibration Curve of MicrobeDiscover.

TABLE S4 | Trimetallic NMs prediction result of available microorganisms.

**Microorganism**	**AuAgPt**	**AuAgPd**	**AuPdPt**	**AgPdPt**	**Average Result**
Aeromonas veronii ATCC 35622	0.50	0.55	0.55	0.52	0.53
Aspergillus carbonarious CICC 2215	0.05	0.10	0.08	0.09	0.08
Aspergillus carbonarious CICC 41252	0.15	0.27	0.23	0.26	0.23
Aspergillus niger CICC 2475	0.71	0.59	0.60	0.55	0.61
Aspergillus niger CICC 40553	0.68	0.58	0.57	0.53	0.59
Bacillus licheniformis ATCC 14580	0.60	0.56	0.59	0.54	0.57
Bacillus licheniformis ATCC 9945a	0.62	0.60	0.61	0.57	0.60
Bacillus licheniformis F11	0.58	0.51	0.52	0.45	0.52
Brevibacterium casei ATCC 35513	0.58	0.58	0.57	0.53	0.57
Escherichia coli DM2516	0.42	0.48	0.47	0.45	0.46
Escherichia coli JE1011	0.41	0.51	0.48	0.48	0.47
Escherichia coli JM103D	0.38	0.48	0.45	0.45	0.44
Klebsiella oxytoca SA2	0.19	0.18	0.19	0.17	0.18
Pantoea agglomerans ATCC 27155	0.71	0.58	0.64	0.56	0.62
Pseudomonas aeruginosa F9676	0.34	0.30	0.29	0.24	0.29
Rhodobacter capsulatus NBRC 16435	0.63	0.56	0.57	0.49	0.56
Rhodobacter sphaeroides ATCC 17023	0.64	0.58	0.59	0.52	0.58
Rhodopseudomonas palustris DSM 5869	0.20	0.33	0.27	0.30	0.27
Rhodopseudomonas palustris H3C	0.47	0.29	0.29	0.24	0.32
Rhodopseudomonas palustris JCM 2524	0.14	0.10	0.11	0.08	0.11
Serratia marcescens ATCC 13880	0.23	0.27	0.26	0.26	0.26
Streptomyces griseus JCM 4626	0.62	0.62	0.61	0.57	0.61
Streptomyces griseus R63	0.62	0.62	0.62	0.58	0.61
Trichoderma viride CICC 13038	0.57	0.58	0.57	0.52	0.56

Continued on next page

BIOSYNTHESIS OF NMS WITH MICROBEDISCOVER

We used *Shewanella oneidensis* MR-1 to synthesize trimetallic materials AuPdPt. XPS spectra (Figure S) reveales that the binding energy of Au 4f spectra (83.8 eV and 87.5 eV) was located at the characteristic position of metal Au, indicating its existence in a metallic state. The binding energies of Pd 3d (336.8 eV and 342 eV, 338.2 eV and 343.4 eV) and Pt 4f (72.6 eV and 75.9 eV , 75.0 eV and 78.3 eV) spectra have undergone a positive shift compared to their metallic states, indicating that they mainly existed in oxidized states (such as PdO, PtO, or hydroxides). The observed O 1s peak in the full spectrum was located at 531.1 eV, which was a typical feature of metal oxides/hydroxides, providing evidence for the surface oxidation of Pd and Pt. The results shows that on the surface of alloy nanoparticles, Pd and Pt, having more active chemical properties, were preferentially oxidized to form a surface oxide layer, while inert Au remained in a metallic state. We also analyzed the particle size, statistics show that the average diameter of the nanoparticles was 18.32 nm $\pm$ 8.54 nm (Fig.S9a). To verify the reproducibility of the synthesis method, we independently conducted synthesis experiments under identical conditions. The average diameters of nanoparticles was 19.91 nm $\pm$ 7.12 nm (Fig.S9b). It should be noted that the particle size distribution of microbial synthesized nanoparticles was relatively wide, which reflects the inherent heterogeneity of the synthesis in biological systems. Our repeated experiments have shown that this method can stably and repetitively prepare nanoparticles with similar size ranges.

We used *Shewanella oneidensis* MR-1 to synthesize trimetallic materials AgPdPt, AuAgPt and AuAgPd. The nanoparticles in Fig.S10 and the corresponding EDS mapping shows that AgPdPt NMs were possibly synthesized on the cell surface of *Shewanella oneidensis* MR-1. The nanoparticles and the corresponding element distribution in Fig.S11 show AuAgPt NMs were possibly synthesised on the cell surface of *Shewanella oneidensis* MR-1. Fig.S12 shows that there were also nanoparticles on *Shewanella oneidensis* MR-1 cell surface, which were consistent with Pd, Au and Ag. It suggests that AuAgPd NMs were possibly synthesized. The above results reveal that *Shewanella oneidensis* MR-1 was able to synthesize these four trimetallic NMs. We evaluated the probability of microbial synthesis of three metal nanoparticles currently available, and the model predicted a relatively high probability of *Pantoea agglomerans* ATCC 27155 and a low probability of *Aspergillus carbonarious* CICC 2215. Thus we verified their performance through synthesis, the result are as shown in Fig.5 and Fig.S11-S17.

We have made predictions about trimetallic materials for different bacterial strain genera, the overview of prediction result is shown in Fig.S20. We also use MicrobeDiscover to predict other uncommon trimetallic materials by adding Rh, Ru and Cu. We use hierarchical clustering based on the prediction result, so patterns of more closely related trimetallic NMs and reported microorganisms would cluster making interpretation more accessible, which is as shown in Fig.S21. The detailed result can be access by trimetallic materials.csv.

2 | Website application with MicrobeDiscover

M2MAnalysis can be divided into Data Source, Server Layer, and Frontend from bottom to top. Employing Docker containerization for deployment facilitates streamlined pr ogram construction, delivery, and execution, concurrently mitigating challenges associated with environmental variations and dependencies. For ease of management, data is stored in Neo4j and MySQL databases, ensuring the security and reliability of data storage and retrieval. The frontend of M2MAnalysis is crafted using the Vue.js framework in conjunction with Apache ECharts and Element Plus libraries, enhancing the overall user experience. Within the Server, the web frontend communicates with the Java-powered backend server through asynchronous HTTP requests, utilizing JSON as the exchange format. This communication is established through a RESTful API implemented with Spring Boot, currently featuring 12 available interfaces. This framework guarantees efficient and effective communication between the frontend and backend, all while upholding the confidentiality and integrity of data.

The M2MAnalysis web server features a simple interface for performing knowledge search and analysis of material synthesis by microorganisms and obtaining related novel microorganisms with research value. Users can search for knowledge, including microorganisms, material and systhesis method, of interest and explore the latest research progress in the field. 1. Knowledge Search Module A user can search knowledge by two ways: 1) by clicking the dataset category; 2) by searching for the keyword in the search bar. For different search contents, M2MAnalysis provides corresponding knowledge detail display pages. 2. Microorganisms details. In this page, M2MAnalysis presents the attribute details, linkage tree,

knowledge network extracted from literature, and related references. 3. Material details. For the type of material, beside attribute detail, knowledge network extracted from literature and related references, the structure diagram is also displayed. **Synthesis method details.** The synthesis method include microorganism, material, precursor and synthesis method description. **Reference details.** The metadata of reference including author, journal, keyword, abstract, etc., is provided in the reference details page.

2.1 | Knowledge Statistical Analysis Module

M2MAnalysis provide two types of knowledge statistical analysis: (1) The knowledge graph which presented a knowledge network related to input, with knowledge categories including microorganisms, materials, generation methods, and precursors; (2) The regional distribution of research which displays the distribution of research areas for input.

2.2 | Microbes Prediction Module

As shown in Fig.S22, in addition to domain knowledge retrieval and overview functions, M2MAnalysis also provides solution analysis and recommendation functions for experimental personnel. The user inputs the target material product elements and microbial species, and M2MAnalysis uses knowledge data to statistically analyze the generation method of microbial synthesis input target element products for this type of microorganism. It also provides prediction and ranking of the probability of other unreported microbial synthesis target products for this type, and provides synthesis scheme suggestions for experimental personnel.

2.3 | Case Study of M2MAnalysis

CdS, as a semiconductor material with visible light absorption, is widely used in photocatalysis. In recent years, in the microbial-material hybrid photosynthetic system, CdS synthesized by microorganisms acted as a photocatalyst to introduce the energy of photons into microorganisms and promoted the catalytic process of microorganisms.In order to expand the application of CdS in material-microbe hybrid systems, it is necessary to know which microorganisms can synthesize CdS. Firstly, the M2Manalysis platform allows a quick search for information about CdS synthesized by microorganisms in the literatures, including which microorganisms have been demonstrated to synthesize CdS, the location of CdS in the microbes, the particle size and shape of the CdS and so on. With this quickly available information, researchers can quickly screen which microbial-CdS combinations are compatible with their requirements. For example, if researchers want to construct a hybrid photosynthetic system in which CdS is located in the periplasmic space of microorganisms, the information on the location of CdS in the platform can be quickly screened to obtain the species of microorganisms.In addition, the platform's unique analytical prediction function will provide information on which microbes can synthesise CdS, which microbes are most likely to synthesise CdS.

TABLE S5 | The Top3 microbes from NCBI of different material.

Material	TOP1	TOP2	TOP3
As_2S_3	*Zoogloea oryzae*	*Dimorphococcus lunatus*	*Enterobacteriaceae bacterium* D57
Bi	*Pichia punctispora*	*Kocuria assamensis*	*Stachybotrys kampalensis*
Bi_2Se_2	*Zoogloea oryzae*	*Pseudodesulfovibrio alkaliphilus*	*Stachybotrys limonispora*
CdSe	*Marinococcus halophilus*	*Dimorphococcus lunatus*	*Candidatus Ruthenibacterium merdipullorum*
CdS	*Hericium bharengense*	*Hericium bembedjaense*	*Sargassum siliquosum*
CdS/Ag	*Condenascus tortuosus*	*Shewanella piezotolerans*	*Sargassum fulvellum*

Continued on next page

TABLE S5 | The Top3 microbes from NCBI of different material.

Material	TOP1	TOP2	TOP3
CdTe	*Hericium americanum*	*Stachybotrys longispora*	*Hericium cf. alpestre* K(M) 107270
CdS_xSe_{1-x}	*Halamphora subacutiuscula*	*Fusarium tumidum*	*Fusarium cf.* bullatum CC06-47S-1_PCNB
Co_3O_4	*Stachybotrys limonispora*	*Stachybotrys phaeophialis*	*Stachybotrys reniformis*
Cu	*Orthodes detracta*	*Halamphora subacutiuscula*	*Fusarium tumidum*
CuS	*Kocuria assamensis*	*Shinella sumterensis*	*Pichia punctispora*
$Cu_{2-x}Se$	*Fusarium tumidum*	*Nectriaceae* sp. osu_besc_4b	*Shewanella piezotolerans*
$Fe_3(PO_4)_2\cdot xH_2O$	*Chaetomiaceae* sp. BESC869b	*Scenedesmaceae* sp. Tow 2/24 P-8w	*Chaetomiaceae* sp. HBL-2018a
Fe_2O_3	*Zoogloea oryzae*	*Thaumatomyia rufa*	*Salibacterium halochares*
$Fe_5HO_8\cdot 4H_2O$	*Zoogloea oryzae*	*Thaumatomyia rufa*	*Brucella rhizosphaerae*
MFe_2O_4	*Orthodes detracta*	*Shewanella chilikensis*	*Shewanella inventionis*
Fe^{2+}	primary endosymbiont of *Pedicinus badii*	*Marasmitruncus massiliensis*	*Chaetomiaceae* sp. BESC869b
Au	*Orthodes detracta*	*Halamphora subacutiuscula*	*Pichia punctispora*
Au-Ag	*Sargassum fulvellum*	*Halamphora subacutiuscula*	*Chaetomorpha* cf. inflata G56
Au-Pd	*Stachybotrys sansevieriae*	*Stachybotrys kampalensis*	*Chaetomorpha* cf. inflata G56
Pb	*Condenascus tortuosus*	*Shewanella insulae*	*Sargassum fulvellum*
PbS	*Condenascus tortuosus*	*Shinella sumterensis*	*Stachybotrys sansevieriae*
$Fe_{1+x}S$	*Orthodes detracta*	*Halamphora*	*Chaetomiaceae* sp. BESC869b
γ-Fe_2O_3	*Chaetomiaceae* sp. BESC869b	*Orthodes detracta*	*Halamphora subacutiuscula*
Fe_3O_4	*Zoogloea oryzae*	*Thaumatomyia rufa*	*Stachybotrys limonispora*
MnO_2	*Salimicrobium luteum*	*Zoogloea oryzae*	*Pseudodesulfovibrio alkaliphilus*
MnO	*Zoogloea oryzae*	*Thaumatomyia rufa*	*Chaetomorpha melagonium*
HgS	*Dimorphococcus lunatus*	*Zoogloea oryzae*	*Marinococcus halophilus*
Pd	*Condenascus tortuosus*	*Stachybotrys sansevieriae*	*Kocuria assamensis*
Pd-Ag	*Condenascus tortuosus*	*Chlorops laetus*	*Escherichia senegalensis*
Pt	*Orthodes detracta*	*Halamphora subacutiuscula*	*Pichia punctispora*

Continued on next page

TABLE S5 | The Top3 microbes from NCBI of different material.

Material	TOP1	TOP2	TOP3
Pd-Pt	*Stachybotrys sansevieriae*	*Stachybotrys kampalensis*	*Chaetomorpha* cf. inflata G56
Sb_2O_3	*Stachybotrys phaeophialis*	*Stachybotrys reniformis*	*Stachybotrys cylindrospora*
$Se_{8-x}S_x$	*Fusarium tumidum*	*Chaetomiaceae* sp. BESC869b	*Marasmitruncus massiliensis*
Se	*Halamphora subacutiuscula*	*Fusarium tumidum*	*Gliocladiopsis tenuis*
$Si\text{-}SiO_2$	Halamphora subacutiuscula	Pichia punctispora	Halamphora bistriata
Ag	Orthodes detracta	Halamphora subacutiuscula	Shewanella piezotolerans
Ag_2S	Stachybotrys sansevieriae	Condenascus tortuosus	Stachybotrys reniformis
SnO_2	Enterobacteriaceae bacterium D57	Salimicrobium luteum	Pseudodesulfovibrio alkaliphilus
Te	Halamphora subacutiuscula	Orthodes detracta	Pichia punctispora
TiO_2	Shinella sumterensis	Sargassum siliquosum	Pichia punctispora
UO_2	Stachybotrys phaeophialis	Stachybotrys reniformis	Stachybotrys kampalensis
ZnO	Scenedesmaceae sp. Tow 2/24 P-8w	Halamphora siqueirosii	Shewanella inventionis
ZnS	Pichia punctispora	Kocuria assamensis	Shinella sumterensis
$Au+Fe_3O_4$	Scenedesmaceae sp. Tow 2/24 P-8w	Thaumatomyia rufa	Lemmermannia komarekii
Pt-Cu	Shewanella piezotolerans	Halamphora subacutiuscula	Pichia punctispora
Cd_4HgS_5	Shewanella piezotolerans	mastodon intestinal bacterium 101.3.37	Condenascus tortuosus
Au-Se	Marasmitruncus massiliensis	Sargassum fulvellum	Halamphora subacutiuscula
Pd-Ru	Condenascus tortuosus	Stachybotrys sansevieriae	Stephanopyxis palmeriana
Pt-Ag	Condenascus tortuosus	Shewanella piezotolerans	Sargassum fulvellum
Ag_2Se	Chaetomiaceae sp. BESC869b	Fusarium tumidum	Candidatus Faecalibacterium faecigallinarum
$Ca_5(PO_4)_3(OH)$	Zoogloea oryzae	Thaumatomyia rufa	Pseudodesulfovibrio alkaliphilus
MnS	Dimorphococcus lunatus	Salimicrobium luteum	Bacillaceae bacterium WB85
NiO	Shewanella inventionis	Shinella sumterensis	Stachybotrys cf. elegans HGUP 0310
CuFe	Orthodes detracta	Halamphora subacutiuscula	Pichia punctispora
$CoFe_2O_4$	Stachybotrys limonispora	Shinella sumterensis	Stachybotrys phaeophialis

Continued on next page

TABLE S5 | The Top3 microbes from NCBI of different material.

Material	TOP1	TOP2	TOP3
Fe_xS_y	Pichia punctispora	Shewanella piezotolerans	Shinella sumterensis
Co_3O_4	Stachybotrys limonispora	Stachybotrys phaeophialis	Pantoea hericii
$BaTiO_3$	Salimicrobium luteum	Halamphora tenuis	Halamphora mosensis
Cd	Neurospora novoguineensis	Mucor chiangraiensis	Halamphora subacutiuscula
Ag/AgCl	Shewanella piezotolerans	Condenascus tortuosus	Marasmitruncus massiliensis
Mg	Condenascus tortuosus	Shewanella piezotolerans	Marasmitruncus massiliensis
MgO	Enterobacteriaceae bacterium D57	Faecalibacterium prausnitzii	Zoogloea oryzae
FeS	Stachybotrys reniformis	Stachybotrys phaeophialis	Stachybotrys sansevieriae
Sb_2S_3	Stachybotrys reniformis	Stachybotrys kampalensis	Pantoea hericii
CuO	Halamphora siqueirosii	Shewanella inventionis	Halamphora cf. fluminensis s0314
FeO	Halamphora siqueirosii	Scenedesmaceae sp. Tow 2/24 P-8w	Shewanella inventionis
Fe	Orthodes detracta	Halamphora subacutiuscula	Pichia punctispora
Rh	Orthodes detracta	Shewanella chilikensis	Pichia punctispora
Ni	Orthodes detracta	Pichia punctispora	Halamphora subacutiuscula
Ru	Orthodes detracta	Kocuria assamensis	Pichia punctispora
Co	Orthodes detracta	Halamphora subacutiuscula	Pichia punctispora
Li	Orthodes detracta	Pichia punctispora	Halamphora subacutiuscula
$CaCO_3$	Pichia punctispora	Halamphora subacutiuscula	Shinella sumterensis
Zr	Gliocladiopsis tenuis	Achaetomium umbonatum	Achaetomium cristalliferum
SiO_2-Ag	Condenascus tortuosus	Shewanella piezotolerans	Neobacillus jeddahensis
Ag_2O	Halamphora subacutiuscula	Chaetomiaceae sp. BESC869b	Fusarium tumidum
$Fe(OH)_3$	Zoogloea oryzae	Thaumatomyia rufa	Chlorops rufinus
$CuAlO_2$	Orthodes detracta	Halamphora subacutiuscula	Fusarium cf. bullatum CC06-47S-1_PCNB
Au-Ag-Cu	Halamphora subacutiuscula	Marasmitruncus massiliensis	Chaetomiaceae sp. BESC869b
Au-Ag-Pt	Enterobacteriaceae bacterium symbiont of Parasaissetia nigra	Marasmitruncus massiliensis	Condenascus tortuosus

Continued on next page

TABLE S5 | The Top3 microbes from NCBI of different material.

Material	TOP1	TOP2	TOP3
Au-Pd-Cu	Stachybotrys kampalensis	Stachybotrys sansevieriae	Chaetomorpha cf. inflata G56
Au-Pd-Pt	Stachybotrys kampalensis	Condenascus tortuosus	Chaetomorpha cf. inflata G56
Au-Cu-Pt	Halamphora subacutiuscula	Marasmitruncus massiliensis	Shewanella piezotolerans
Ag-Pd-Cu	Dimorphococcus lunatus	Enterobacteriaceae bacterium D57	Condenascus tortuosus
Ag-Pd-Pt	Dimorphococcus lunatus	Stachybotrys sansevieriae	Enterobacteriaceae bacterium D57
Ag-Cu-Pt	Marasmitruncus massiliensis	Shewanella piezotolerans	Condenascus tortuosus
Pd-Cu-Pt	Stachybotrys sansevieriae	Zoogloea oryzae	Stachybotrys kampalensis
Au-Pt	Condenascus tortuosus	Enterobacteriaceae bacterium symbiont of Parasaissetia nigra	Marasmitruncus massiliensis
Ag-Pd	Dimorphococcus lunatus	Enterobacteriaceae bacterium D57	Enterobacteriaceae bacterium R9.62
Ag-Pt	Condenascus tortuosus	Enterobacteriaceae bacterium D57	Enterobacteriaceae bacterium symbiont of Parasaissetia nigra
Au-Ag-Pd	Dimorphococcus lunatus	Enterobacteriaceae bacterium D57	Condenascus tortuosus

Continued on next page

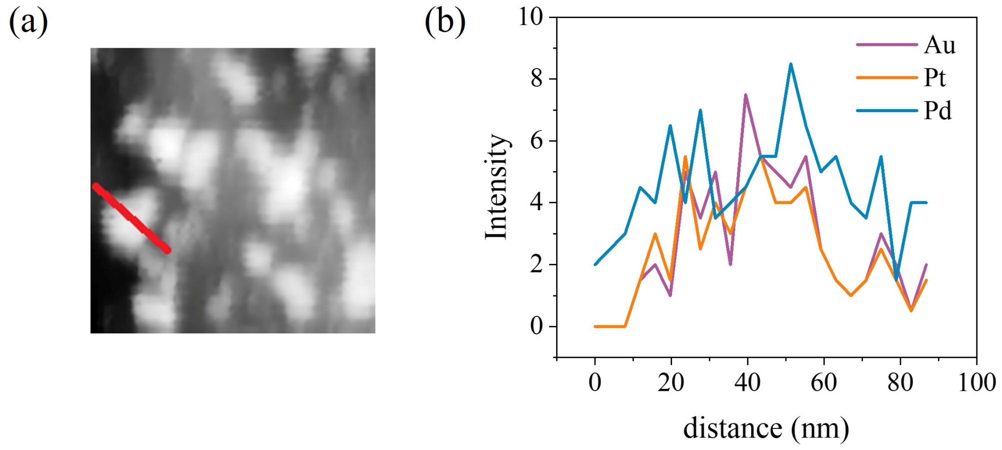

FIGURE S7 | STEM-EDS line scan analysis of AuPdPt trimetallic nanoparticles. (a) The position of the EDS line scan. (b) The intensity of the three elements in the scanning position.

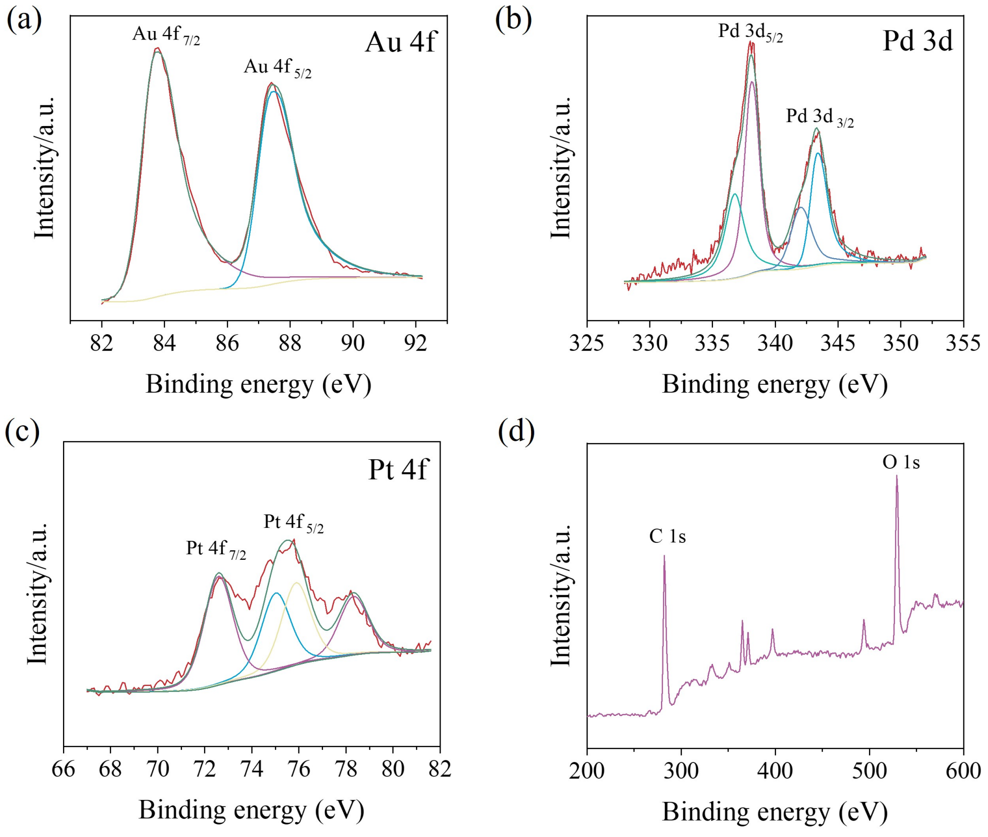

FIGURE S8 | XPS analysis of AuPdPt trimetallic nanoparticles.

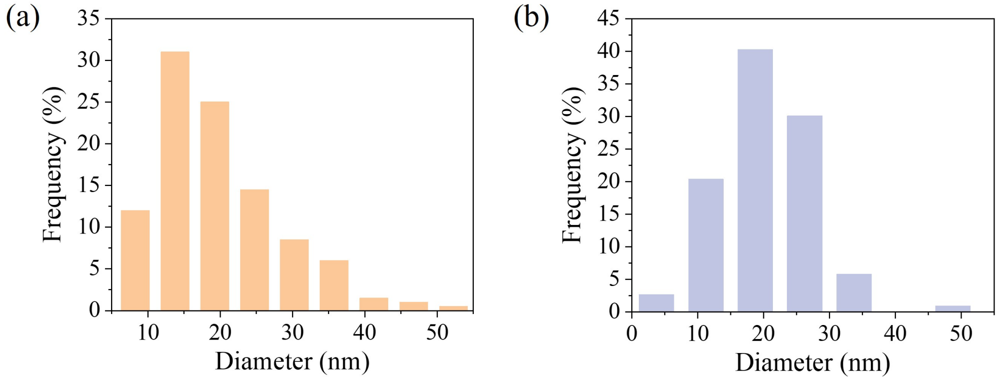

FIGURE S9 | Statistical particle size data of two AuPdPt trimetallic nanoparticles synthesis experiments.

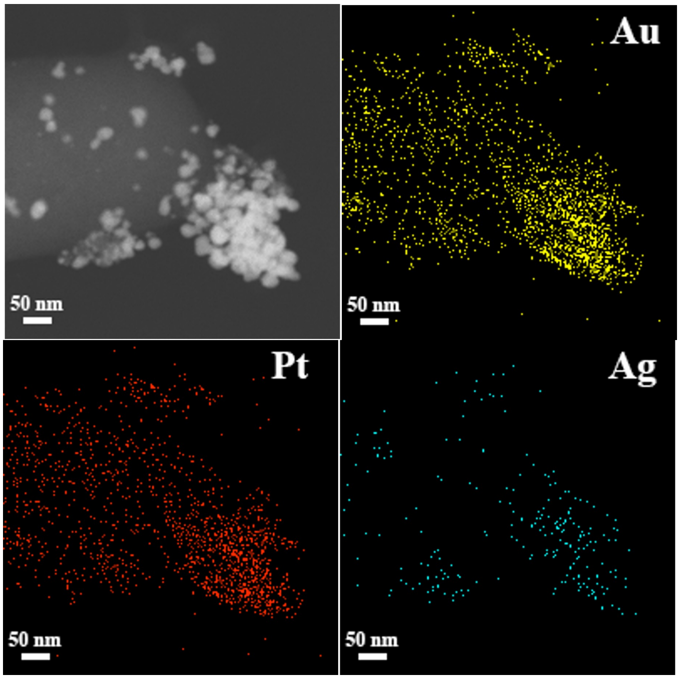

FIGURE S10 | TEM and EDS mapping images of *Shewanella oneidensis* MR-1, after the synthesis of PtAuAg trimetallic NMs.

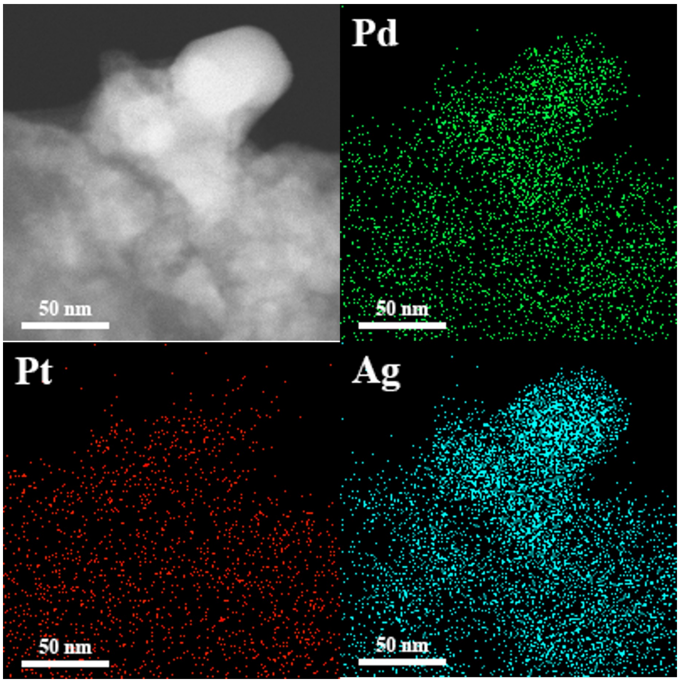

FIGURE S11 | TEM and EDS mapping images of *Shewanella oneidensis* MR-1, after the synthesis of PdAgPt trimetallic NMs.

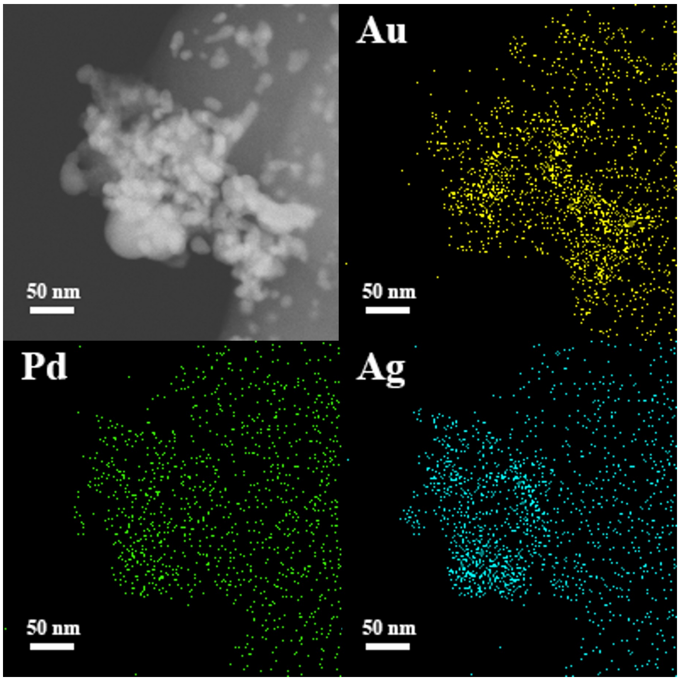

FIGURE S12 | TEM and EDS mapping images of *Shewanella oneidensis* MR-1, after the synthesis of PdAuAg trimetallic NMs.

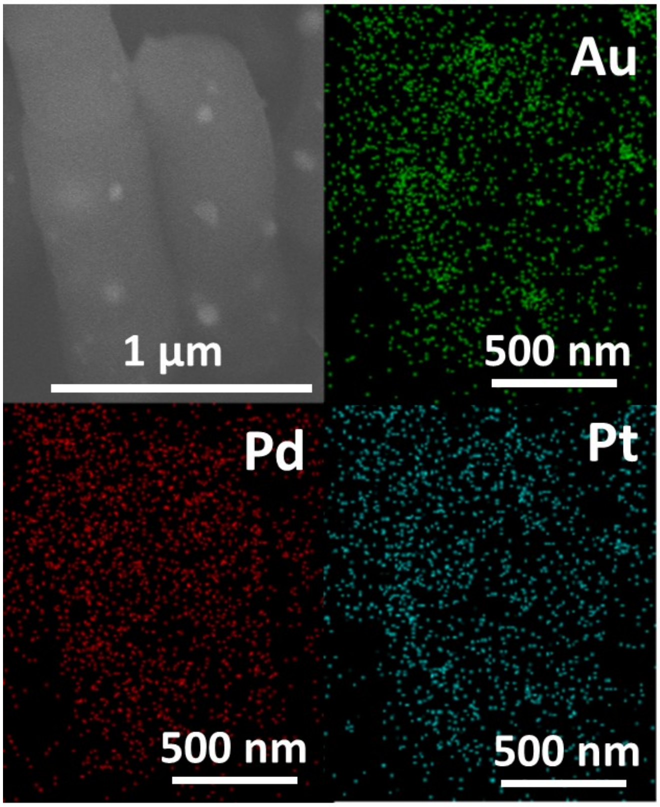

F I G U R E S13 | SEM and EDS mapping images of *Pantoea agglomerans* ATCC 27155 after the synthesis of AuPdPt trimetallic NMs.

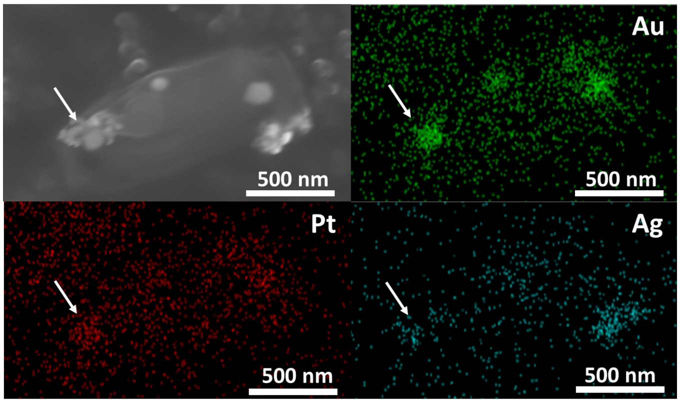

F I G U R E S14 | SEM and EDS mapping images of *Pantoea agglomerans* ATCC 27155, after the synthesis of AuPtAg trimetallic NMs.

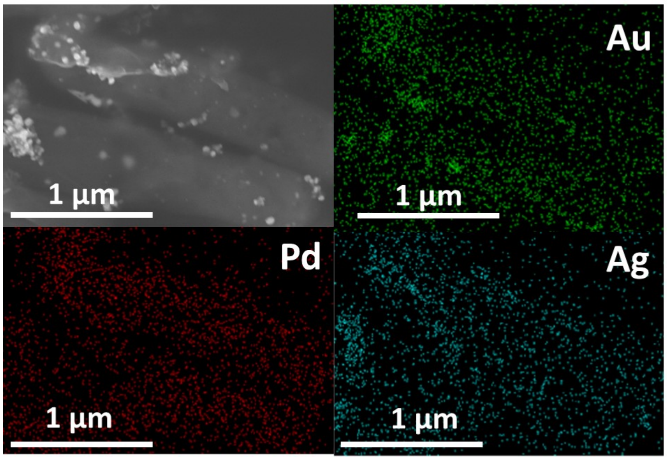

FIGURE S15 | SEM and EDS mapping images of *Pantoea agglomerans* ATCC 27155, after the synthesis of AuPdAg trimetallic NMs.

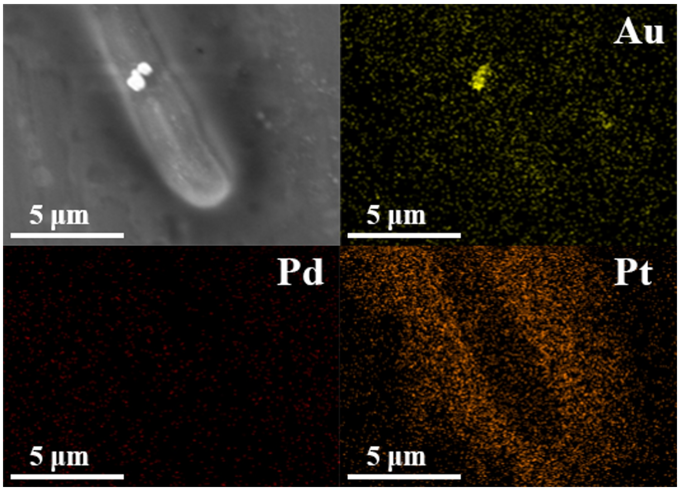

FIGURE S16 | SEM and EDS mapping images of *Aspergillus carbonarious* CICC 2215, after the synthesis of AuPdPt trimetallic NMs.

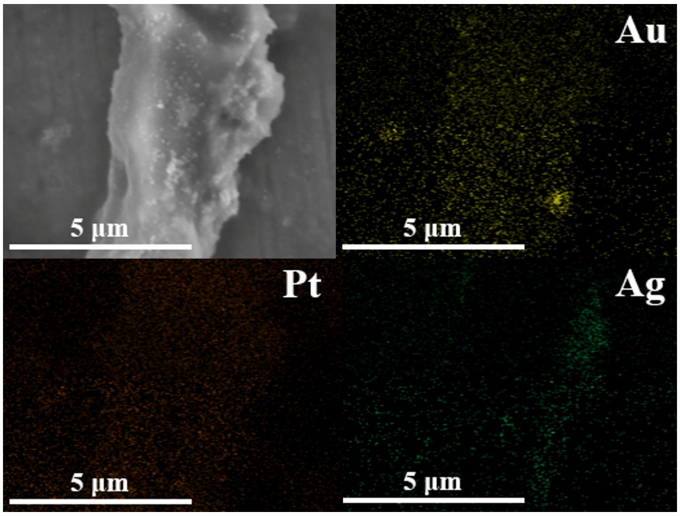

FIGURE S17 | SEM and EDS mapping images of *Aspergillus carbonarious* CICC 2215, after the synthesis of AuPtAg trimetallic NMs.

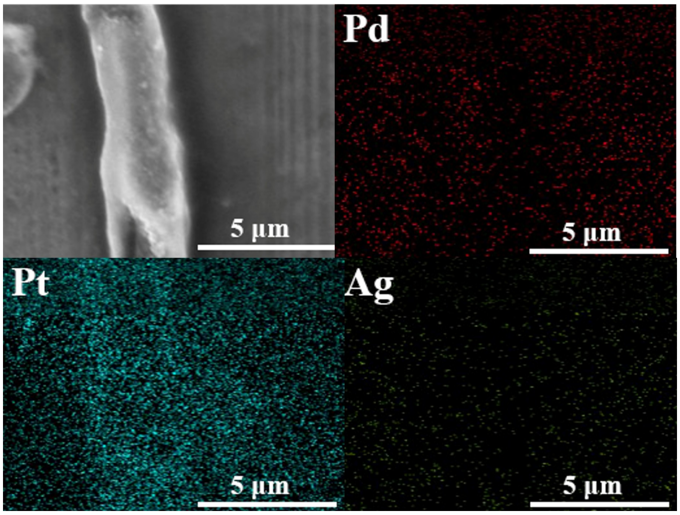

FIGURE S18 | SEM and EDS mapping images of *Aspergillus carbonarious* CICC 2215, after the synthesis of PdPtAg trimetallic NMs.

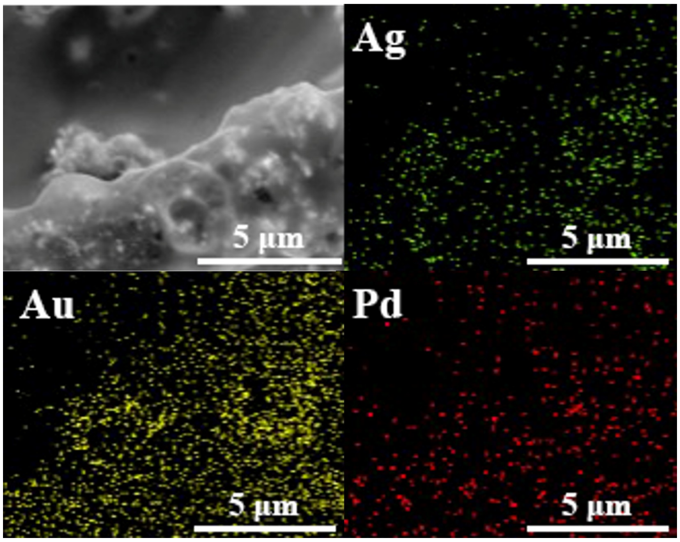

FIGURE S19 | SEM and EDS mapping images of *Aspergillus carbonarious* CICC 2215, after the synthesis of AuPdAg trimetallic NMs.

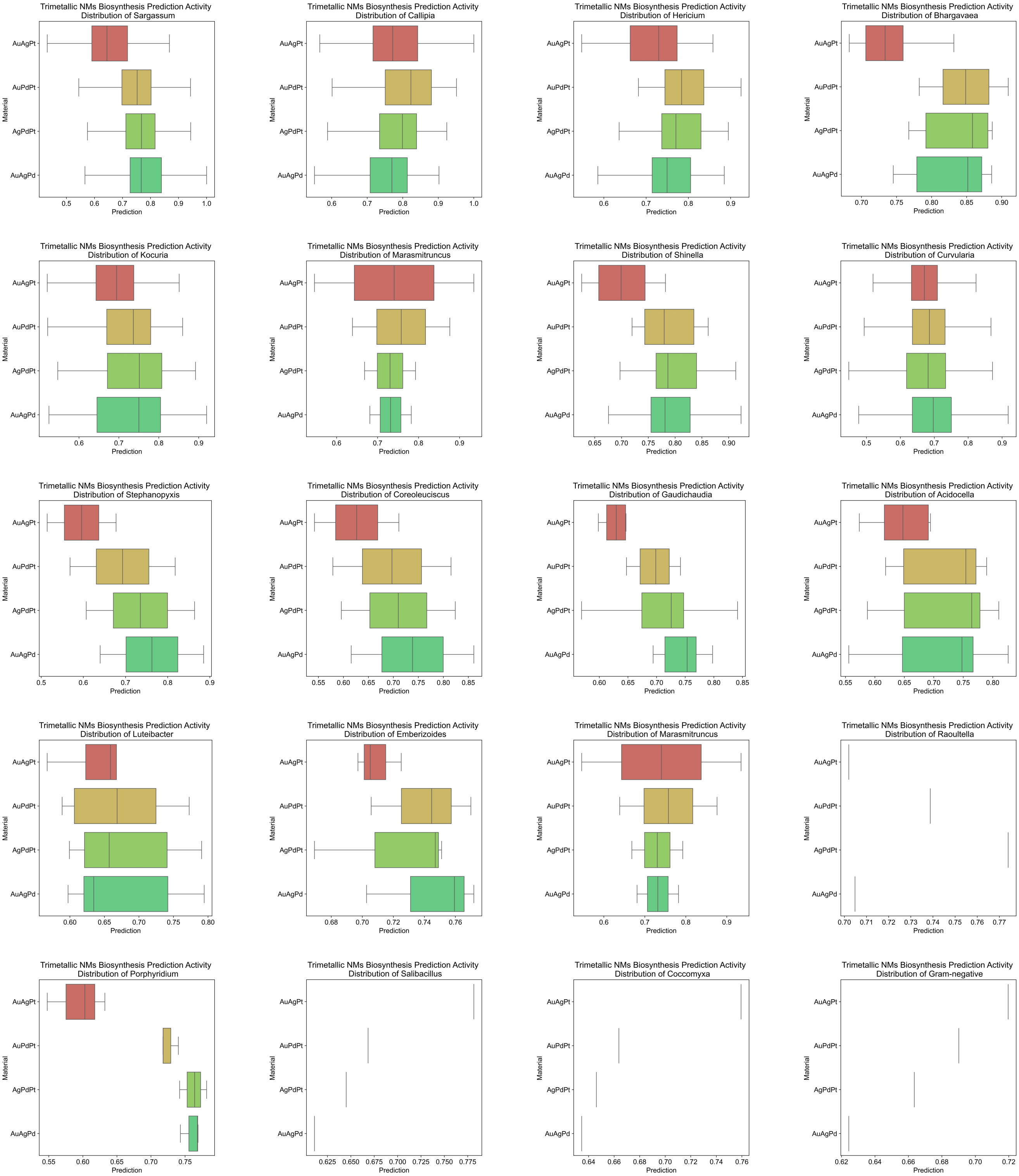

FIGURE S20 | Predictions about trimetallic materials for different bacterial strain genera (Top 20).

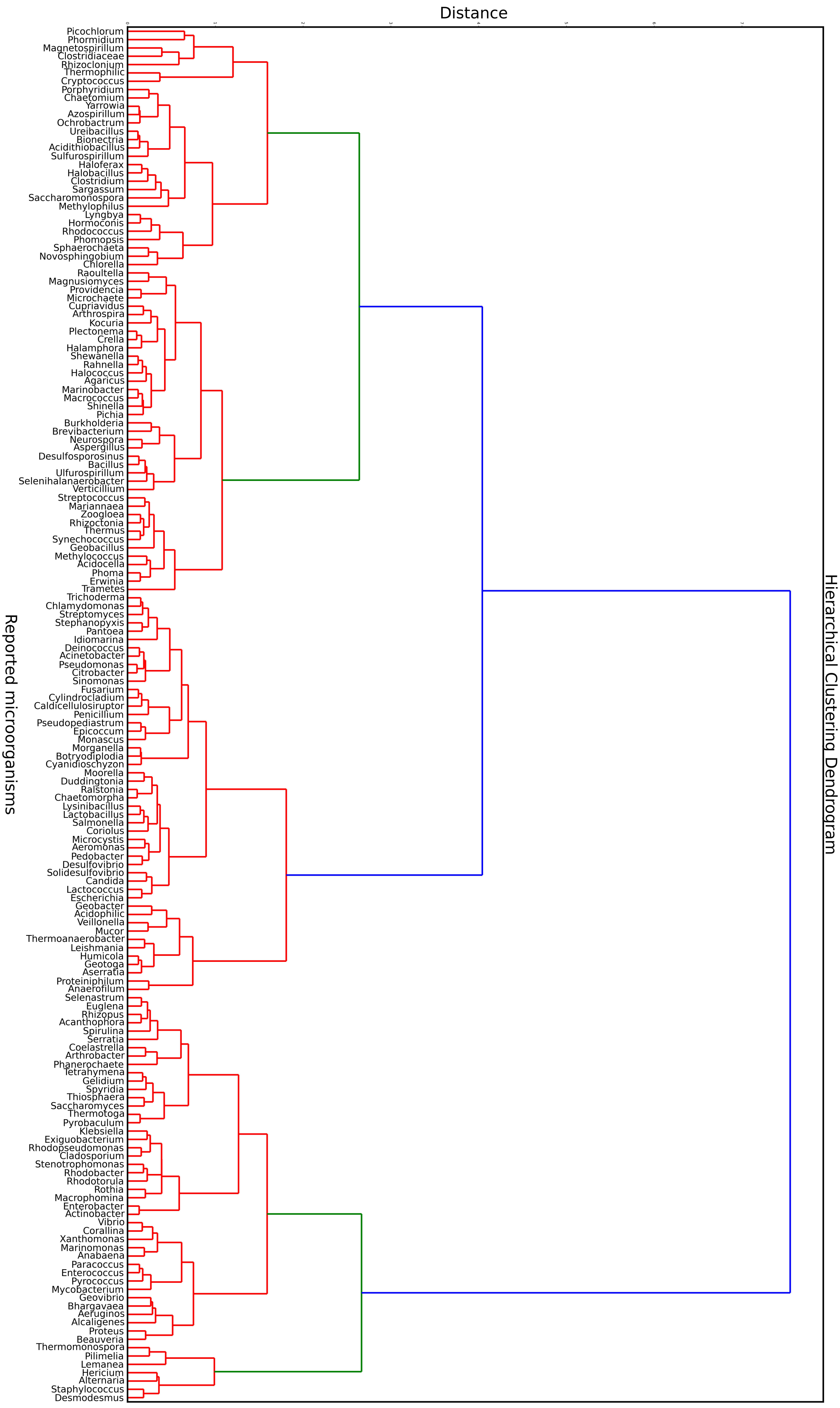

FIGURE S21 | Hierarchical clustering result of reported microorganisms, strains that tend to synthesize substances similar to trimetals will be clustered together.

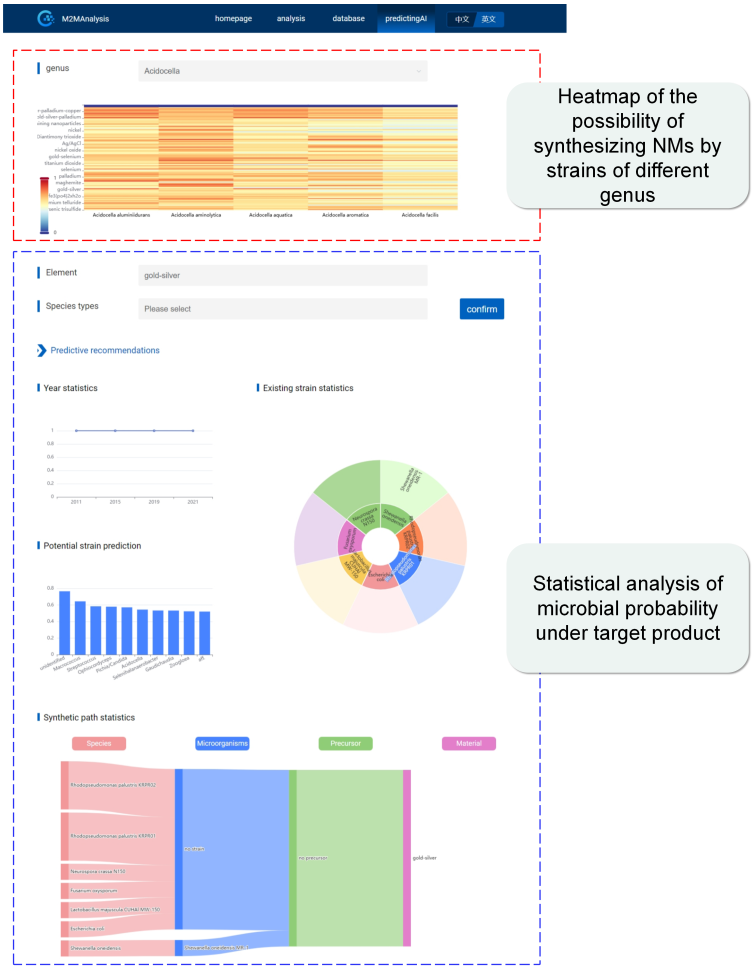

FIGURE S22 | Microbes Prediction Module of M2MAnalysis.

References

1. Yi Du, Ludi Wang, Mengyi Huang, Dongze Song, Wenjuan Cui,, and Yuanchun Zhou. 2023. “Autodive: An Integrated Onsite Scientific Literature Annotation Tool.” In *Annual Meeting of the Association for Computational Linguistics*, .
2. Zachary N Flamholz, Steven J Biller,, and Libusha Kelly. “Large language models improve annotation of prokaryotic viral proteins.” *Nature Microbiology* 9, no. 2 (2024): 537–549.
3. Junyoung Chung, Çaglar Gülçehre, KyungHyun Cho,, and Yoshua Bengio. “Empirical Evaluation of Gated Recurrent Neural Networks on Sequence Modeling.” *CoRR*abs/1412.3555.
4. Michael Schlichtkrull, Thomas Kipf, Peter Bloem, Rianne Berg, Ivan Titov,, and Max Welling. “Modeling Relational Data with Graph Convolutional Networks.” .
5. Yuxiao Dong, Nitesh V. Chawla,, and Ananthram Swami. 2017. “metapath2vec: Scalable Representation Learning for Heterogeneous Networks.” In *Proceedings of the 23rd ACM SIGKDD International Conference on Knowledge Discovery and Data Mining*, 135–144. ACM.
6. Thomas N. Kipf, and Max Welling. 2017. “Semi-Supervised Classification with Graph Convolutional Networks.” In *Proceedings of the International Conference on Learning Representations (ICLR)*, .
7. Petar Veličković, Guillem Cucurull, Arantxa Casanova, Adriana Romero, Pietro Liò,, and Yoshua Bengio. 2018. “Graph Attention Networks.” In *Proceedings of the 6th International Conference on Learning Representations (ICLR)*, .
8. Junyoung Chung, Caglar Gulcehre, Kyunghyun Cho,, and Yoshua Bengio. “Empirical evaluation of gated recurrent neural networks on sequence modeling.” . *arXiv preprint arXiv:1412.3555.*
9. Sepp Hochreiter, and Jürgen Schmidhuber. “Long short-term memory.” *Neural Computation* 9, no. 8 (1997): 1735–1780.
10. Tao Qin, Tie-Yan Liu,, and Hang Li. “A general approximation framework for direct optimization of information retrieval measures.” *Inf. Retr.* 13, no. 4 (2010): 375–397.
